# Supplementary material for: Migrasome formation is initiated preferentially in tubular junctions by membrane tension
Source: Biophys J. 2025 Jan 3;124(4):604–19. doi: 10.1016/j.bpj.2024.12.029 (PMC11900186; doi:10.1016/j.bpj.2024.12.029)
Supplement: Document S1. Figures S1 and S2 [file mmc1.pdf]

Biophysical Journal, Volume *124*

Supplemental information

Migrasome formation is initiated preferentially in tubular junctions by membrane tension

Ben Zucker, Raviv Dharan, Dongju Wang, Li Yu, Raya Sorkin, and Michael M. Kozlov

Supplementary Information

Supplementary Video legend

3-tube junction formation followed by swelling formation. A video of formation of 3-tube junction pulled from aspirated GPMV dyed with DiI-C12. Next the aspiration pressure was reduced to zero and then it was increased instantaneously to 0.39 mbar (corresponding to 0.074 mN/m). The video was composed of confocal fluorescence microscopy images of the experiment shown in Figure 1b. The fluorescence intensity is presented in logarithmic scale.

Supplementary Figure S1

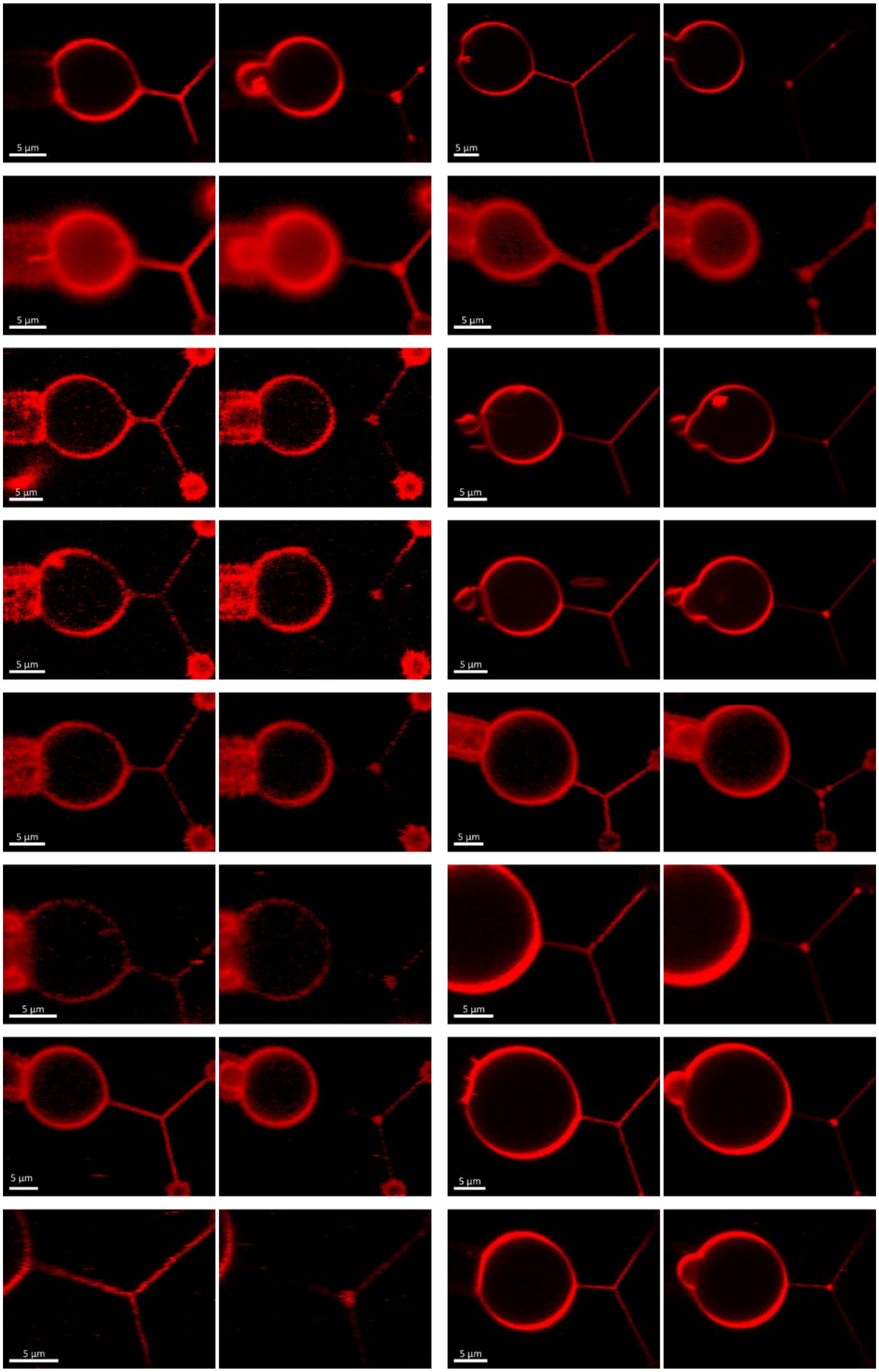

Figure S1. Confocal microscopy images of bulge formation induced by abrupt tension increase in 10 GPMVs dyed with DiI-C12. The images show 16 independent realizations of the system of three membrane tubules pulled out of an aspirated GPMV and connected by a three-way junction. In each panel the left and right images show, respectively, the system before and after the abrupt tension application. In all 16 experiments, the main bulging occurred in the junction.

Supplementary Information

Bulge coexistence in junction and tubular arms

The master curves obtained for the cylinder and the junction systems (Fig.7C of the main part) enable analysis of a mixed state of the junction system in which bulging occurs in parallel in the junction and in the cylindrical part of a tubular arm. Our goal here is to demonstrate that such coexistence of bulges represents a (quasi) equilibrium and to analyze the stability of this state with respect to bulging only in the junction or only in the tubular arm.

As above, in the initial state the junction is under a tension γ_0 and a vanishing pressure, $P_0 = 0$, the arm length, L_0, and the corresponding initial area, A_0, and volume, V_0. We assume L_0 to be so long that beginning from a distance L_j from the junction center the shape of each arm is unperturbed by the junction and, therefore, must behave as a one-cylindrical system. We consider the part of one of the arms spanned between L_j to L_0 as a one-cylindrical subsystem of the junction system.

We analyze a specific alteration of the whole system consisting in introduction of an extra volume, ΔV , and the following system evolution to the energetically preferable configuration by keeping constant the total area, A_0, the total volume, $V_0 + \Delta V$, and the total arm length, $3L_0$, The total volume addition, ΔV, is taken sufficiently large for driving the bulging transition.

We considered three possible scenarios: bulging only within the junction, bulging only within the one-cylindrical sub-system, parallel bulging in the junction and the one-cylinder sub-system. Our goal was to analyze the parameters and, specifically, the pressure, P, and the tension, γ of the energetically preferable configuration forming in each scenario as a function of the relative volume alteration, $\frac{\Delta V}{V_0}$. The scenario with the lowest pressure, P, for a given $\frac{\Delta V}{V_0}$ was considered as the optimal one.

For the scenario of bulging only in the junction the determination of the parameters of the energetically favorable configuration including the function $P\left(\frac{\Delta V}{V_0}\right)$ were determined according to the recipe described above (Eqs.21-25 of the main part) and based on the master curves for the junction system (Fig.7C red dots).

The procedure for the bulge coexistence scenario or only in the one-cylinder subsystem connected to a junction was a little more involved because of an apriori unknown distribution of the extra-volume ΔV and the related excess volume, V_{exc}, and area, A_{exc}, between the one-cylinder subsystem and the rest of the junction system. The solution in this case consisted in the following steps. We first assumed the excess values in the one-cylinder subsystem and the rest of the junction system to be $V_{exc}^{(cyl)}$, $A_{exc}^{(cyl)}$, and $V_{exc}^{(jun)}$, $A_{exc}^{(jun)}$, respectively. Using this assumption, we found the parameters of bulging, or the sub-bulging swelling, separately for the one-cylinder sub-system and in the rest of the junction system using the above protocols. Then we obtain all the four parameters, $V_{exc}^{(jun)}$, $A_{exc}^{(jun)}$, $V_{exc}^{(cyl)}$, $A_{exc}^{(cyl)}$ as functions of $\frac{\Delta V}{V_0}$ by using the conditions of conservation of the total volume and area of the whole system and the conditions of equal pressure, P, and tension, γ, in the one-cylinder sub-system and the rest of the junction system. The essence of the condition of the pressure and tension equality is the thermodynamic equilibrium between the one-cylider sub-system and the rest of the junction system and, hence, the (quasi) equilibrium of the whole junction system.

It was possible to obtain the numerical solution for the parameters of the coexistence scenarios meaning that the coexistence of bulges in the junction and tubular arm can indeed be a (quasi)equilibrium state of the system.

The computed dependences of pressure on the relative added volume, $P\left(\frac{\Delta V}{V_0}\right)$, are presented for all the scenarios in (Fig.S2A), which shows that bulging in junction only is characterized by the lowest pressure and, hence, must be the most energetically favorable.

In addition, our computations enabled determination of the partitioning of the system's volume between the bulges and the tubular regions, whose results are presented in (Fig.S2 B-D). Finally, the computed conformation of the quasi-equilibrium configuration of coexisting bulges is presented in (Fig.S2 E).

Figure S2

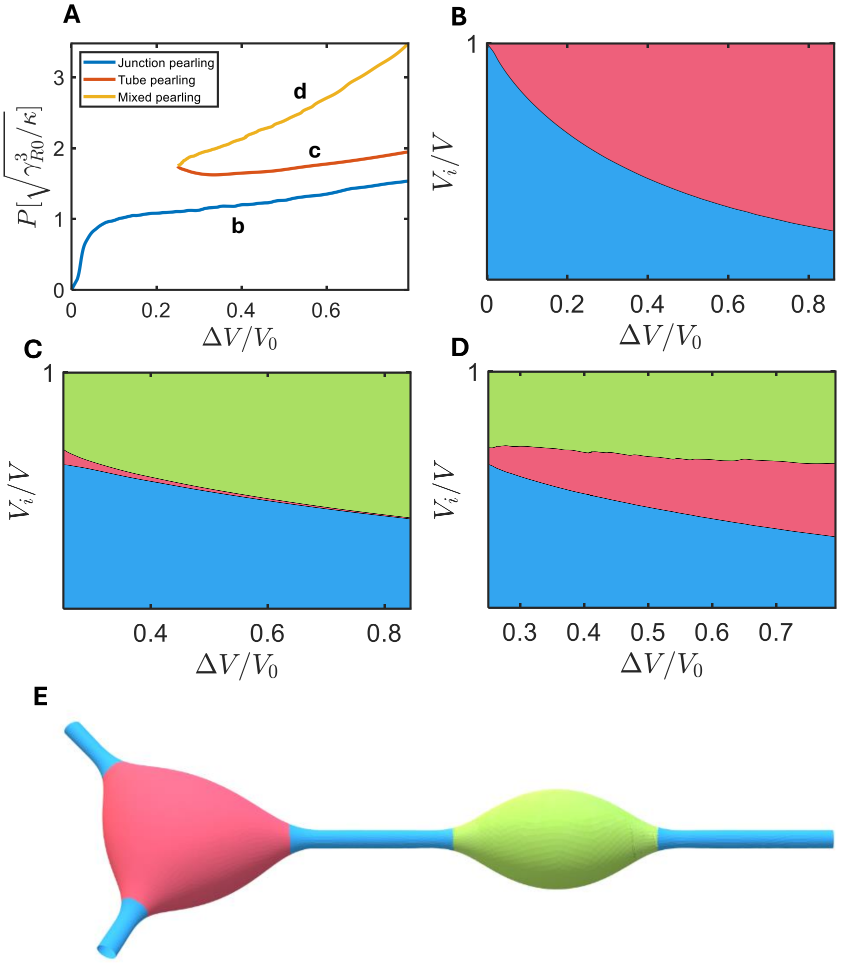

Figure S2. Comparison of bulging scenarios. Computational examination of three scenarios of bulging. (A) The dimensionless pressure, P, of the energetically favorable configurations computed for bulging of the junction (blue curve), bulging of the tubular arm (orange curve), bulging of both the junction and the tubular arm (yellow curve) as functions of the normalized extra volume, $\frac{\Delta V}{V_0}$, where V_0 is the initial volume

of the system. (B-D) Partitioning of volume between different parts of the system in dependence on $\frac{\Delta V}{V_0}$. The volume fraction is denoted by $\frac{V_i}{V}$. The blue, red and green colors correspond respectively to the tubular arms, junctional bulge, and tubular bulge. (B) Bulge in junction. (C) Bulge in tubular arm. (D) Coexistence of bulges in the junction and tubular arm. (E) Example of a computed conformation of the state of coexisting bulges. The length of the tubular network for (A-D) is 16 λ_0.

References

(1) Zucker, B.; Golani, G.; Kozlov, M. M. Model for ring closure in ER tubular network dynamics. *Biophys J* **2023**, *122* (11), 1974-1984. DOI: 10.1016/j.bpj.2022.10.005 From NLM Medline.
(2) Kozlov, M. M. Some aspects of membrane elasticity. In *Soft condensed matter physics in molecular and cell biology*, Poon, W. C. K., Andelman, D. Eds.; Scottish Graduate Series, CRC press, Taylor and Francis group, 2006; pp 79-93.
(3) Gibbs, J. W. *The scientific papers*; Dover, 1961.
(4) Helfrich, W. Elastic properties of lipid bilayers: theory and possible experiments. *Z Naturforsch C* **1973**, *28* (11), 693-703. DOI: 10.1515/znc-1973-11-1209 From NLM Medline.
(5) Brakke, K. A. The Surface Evolver. *Experimental Mathematics* **1992**, *1* (2), 141-165. DOI: 10.1080/10586458.1992.10504253.
