# Supplementary material for: Migrasome formation is initiated preferentially in tubular junctions by membrane tension
Source: Biophys J. 2025 Jan 3;124(4):604–19. doi: 10.1016/j.bpj.2024.12.029 (PMC11900186; doi:10.1016/j.bpj.2024.12.029)
Supplement: Document S2. Article plus supporting material [file mmc3.pdf]

Biophysical Journal
Article

Biophysical Society

Migrasome formation is initiated preferentially in tubular junctions by membrane tension

Ben Zucker,[1,2] Raviv Dharan,[3,2] Dongju Wang,[4,5] Li Yu,[4,5,*] Raya Sorkin,[2,3,*] and Michael M. Kozlov[1,2,*]
[1]Department of Physiology and Pharmacology, Faculty of Medical and Health Sciences, Tel Aviv University, Tel Aviv, Israel; [2]Center for Physics and Chemistry of Living Systems, Tel Aviv University, Tel Aviv, Israel; [3]School of Chemistry, Faculty of Exact Sciences, Tel Aviv University, Tel Aviv, Israel; [4]The State Key Laboratory of Membrane Biology, Tsinghua University-Peking University Joint Center for Life Sciences, School of Life Sciences, Tsinghua University, Beijing, China; and [5]Beijing Frontier Research Center for Biological Structure, Tsinghua University, Beijing, China

ABSTRACT Migrasomes, the vesicle-like membrane microstructures, arise on the retraction fibers (RFs), the branched nanotubules pulled out of cell plasma membranes during cell migration and shaped by membrane tension. Migrasomes form in two steps: a local RF bulging is followed by a protein-dependent stabilization of the emerging spherical bulge. Here, we addressed theoretically and experimentally the previously unexplored mechanism of bulging of membrane tubular systems. We assumed that the bulging could be driven by increases in membrane tension and experimentally verified this hypothesis in live-cell and biomimetic systems. We exposed RF-generating live cells to a hypotonic medium, which produced water flows into the cells and a related increase in the membrane tension. We observed the formation of migrasome-like bulges with a preferential location in the RF branching sites. Next, we developed a biomimetic system of three membrane tubules pulled out of a giant plasma membrane vesicle (GPMV), connected by a junction, and subjected to pulling forces controlled by the GPMV membrane tension. An abrupt increase in the GPMV tension resulted in the generation of migrasome-like bulges mainly in the junctions. To understand the physical forces behind these observations, we considered theoretically the mechanical energy of a membrane system consisting of a three-way tubular junction with emerging tubular arms subjected to membrane tension. Substantiating our experimental observations, the energy minimization predicted a tension increase to drive the formation of membrane bulges, preferably in the junction site, independently of the way of the tension application. We generalized the model to derive universal criteria of bulging in branched membrane tubules.

SIGNIFICANCE The significance of this study is twofold, it proposes a mechanism of the early stage of a biological phenomenon of migrasome formation and analyses the physics behind the shape transformation of branched membrane tubules. While the mechanism of the migrasome maturation was previously considered, the factors determining the nucleation and initial growth of migrasomes remained unknown. Here, we propose the membrane tension as a force leading to the initialization of the migrasome formation. We substantiate this proposal by experiments in live-cell and biomimetic systems and by theoretical modeling. We generalize the model to address the phenomenon of the bulging transition in a system of membrane tubules connected by three-way junctions upon various thermodynamic conditions. Revealing the physical forces driving the migrasome formation is of primary importance for discovering the proteins driving this process and designing the biotechnological strategies for the manipulation of the migrasome generation and cargo loading.

INTRODUCTION

Migrasomes are the recently discovered transient cell organelles whose biogenesis and evolution are tightly related to cell migration (1). Cells crawling on extracellular substrates leave behind retraction fibers (RFs), tens of microns long and about 100-nm-thick membrane nanotubules pulled out of the cell rear edges. RFs are anchored to the substrates at their tips and in discrete locations along their lengths (2). Importantly, most of the RFs are extensively branched

Submitted October 2, 2024, and accepted for publication December 27, 2024.

*Correspondence: liyulab@mail.tsinghua.edu.cn or rsorkin@tauex.tau.ac.il or michk@tauex.tau.ac.il

Ben Zucker, Raviv Dharan, and Dongju Wang contributed equally to this work.

Editor: Edward Lyman.

https://doi.org/10.1016/j.bpj.2024.12.029

© 2025 The Authors. Published by Elsevier Inc. on behalf of Biophysical Society.
This is an open access article under the CC BY license (http://creativecommons.org/licenses/by/4.0/).

with splitting sites represented by symmetric three-way tubular junctions (1). From the physics perspective, the RF formation is analogous to that of tubular membrane tethers generated by localized application of pulling forces to giant lipid vesicles (3–5). Similarly to the membrane tethers, the membrane tension sets the RF tubular shapes and cross-sectional radii. Migrasome formation and maturation are initiated by local swellings of the RFs into micron-scale spherical bulges. The RFs degrade with time, which results in migrasome separation from cell bodies.

Evidence is being accumulated of the multiple essential functions of migrasomes related to their ability to encapsulate and transport a large variety of cargoes. Migrasomes are involved in such processes as morphogenetic signaling (6), cell-cell communication (6,7), intercellular transport of mRNA and proteins (7), removal from cells of damaged mitochondria (8), spreading of diseases including cancer metastasis (9,10), and the pathogenesis of brain injury (11).

Understanding the fundamentals of migrasome biogenesis and, importantly, the development of strategies for migrasome usage for therapeutic and diagnostic purposes require knowledge of molecules and physical forces involved in the migrasome shaping (12). The established crucial molecules are cholesterol and the specific members of the tetraspanin (TSPAN) family of proteins (13) such as TSPAN4 and/or TSPAN7 (12). A suggested mechanism of the migrasome shaping assumed formation within the RFs of macroscopic membrane domains enriched in TSPAN and cholesterol. A large mechanical stiffness of these domains was proposed to drive the local RF bulging (12).

A recent study provided further phenomenological insights into the mechanism of migrasome formation. It was shown in cells and biomimetic systems that migrasomes emerge in two steps (14). First, local vesicle-like bulges arise on the RFs. While the bulges have been observed to form at different positions along the RFs, their preferable locations were the RF tips and junction sites. This is followed by the second step in which the bulges grow and mature into stable migrasomes through a strong enrichment in TSPAN (14). In case TSPAN was absent from the system, the bulges that emerged in the first step quickly disappeared and the RF membranes recovered their initial configuration (14). This suggests that the major role of TSPAN is in the stabilization of the migrasome structure (12), while the initial local bulging of the RF membrane tubules can be driven by other and, possibly, unspecific factors such as mechanical forces.

The goal of this study is to advance the understanding of migrasome biogenesis by addressing the mechanisms of the first TSPAN-independent step of this process. Using live cells and a biomimetic model system of membrane tethers pulled out of giant plasma membrane vesicles (GPMVs) we demonstrate that the initial bulges can be produced by alterations of the membrane tension, a major factor responsible for RF formation that is also involved in the regulation of numerous intracellular processes mediated by membrane shaping and remodeling (15). We show that the tension-driven nascent migrasomes appear preferentially on tubular junctions, while those formed on tubules are unstable and tend to migrate toward and merge with the junctions. Using the theory of membrane elasticity, we elaborate on the criteria of bulge generation in tubular junctions and tubules upon alterations of the membrane tension. We further generalize the theoretical consideration to derive universal criteria of the bulging of branched membrane tubules. We demonstrate that the tubular junctions are mechanically more susceptible to bulging than the tubules themselves, making the tubular branching sites the most favorable locations for migrasome formation.

MATERIALS AND METHODS

Cell culture

L929 cells (for cell imaging) or HEK293T cells (for biomimetic system) were cultured at 37°C, 5% CO_2 in DMEM (Gibco, Brooklyn, NY, United States) high-glucose medium supplemented with 10% fetal bovine serum (Biological Industries, Beit Haemek, Israel), 1% GlutaMAX (Gibco, Brooklyn, NY, United States), and 1% penicillin-streptomycin (15140122 Gibgo-Thermo Fisher scientific, Waltham, MA, United States).

Live cell imaging

Cells were seeded in a glass-bottom dish (D35C4-20-1.5-N, Cellvis, Mountain View, CA,United States) and allowed to grow for 14–18 h. The dish was precoated with 10 μg/mL fibronectin (F0895, Sigma-Aldrich, Saint Louis, MO, United States) at 37°C to enhance cell attachment. Before imaging, culture medium was gently replaced with a staining solution (2 μg/mL WGA Alexa Fluor 488 conjugate [W11216, Invitrogen, Carlsbad, CA, United States] in DPBS) and cells were incubated for 10 min to achieve bright labeling of plasma membrane. Cells were then transferred to a Nikon A1 laser scanning confocal microscope equipped with a live-cell system. For all experiments, a 5 min 3D time-lapse series was collected to fully capture the progress of swelling. Images were captured with an 8-s interval, at each time point a Z stack containing three slices with 500 nm step size was recorded. Real-time hypotonic stimulation during time-lapse imaging was achieved by using a home-made buffer exchange equipment (16). In brief, two silicon microinjection tubes were connected to two 1 mL syringes after the removal of their sharp needles. One syringe was kept empty, while the other was filled with the desired hypotonic buffer. To introduce a real-time buffer exchange, two holes were created on the lid of a confocal chamber, allowing the penetration of microinjection tubes. During time-lapse imaging, the reservoir solution was aspirated using the empty syringe, and the hypotonic buffer was simultaneously injected from the other syringe.

GPMV formation

GPMVs were produced according to a published protocol (17). In brief, HEK293T cells were stained with 2 μg/mL DiI-C12 (Invitrogen) membrane dye by 10 min incubation, washed with GPMV buffer (20 mM HEPES, 150 mM NaCl, 2 mM $CaCl_2$ [pH 7.4]) twice, and incubated overnight with 1 mL of GPMV buffer containing 1.9 mM DTT (Sigma) and 27.6 mM formaldehyde (Sigma). Secreted GPMVs were then collected and isolated from the cells and immediately used for optical trapping experiments.

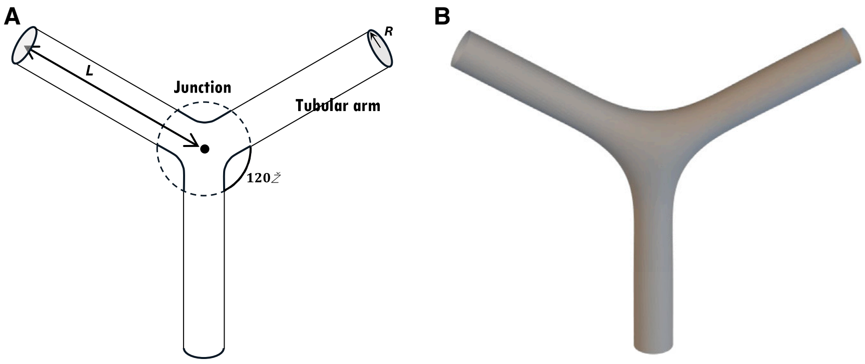

FIGURE 1 A theoretical model system of three tubular arms with a junction. (*A*) Three tubular arms are connected via a three-way junction. The length of each arm is determined as the distance from the junction center to the arm's end and is denoted by *L*. The cross-sectional radius of the arm's ends is denoted by *R*. (*B*) Computed configuration of the system. The computation was performed using the Brakke's Surface Evolver (based on the method of finite elements) for a membrane system characterized by bending rigidity, κ, tension, γ, and a vanishing luminal pressure $P = 0$.

Optical Tweezers Setup

The experiments were performed using a C-trap confocal fluorescence optical tweezers setup (LUMICKS, Amsterdam, the Netherlands) made of an inverted microscope based on a water immersion objective (NA 1.2) together with a condenser top lens placed above the flow cell. The optical traps are generated by splitting a 10 W 1064-nm laser into two orthogonally polarized, independently steerable optical traps. To steer the two traps, one coarse-positioning piezo stepper mirror and one accurate piezo mirror were used. Optical traps were used to capture polystyrene microbeads. The displacement of the trapped beads from the center of the trap was measured and converted into a force signal by back-focal-plane interferometry of the condenser lens using two position-sensitive detectors. The samples were illuminated by a bright-field 850-nm LED and imaged in transmission onto a metal-oxide semiconductor (CMOS) camera.

Confocal fluorescence microscopy

The C-Trap uses a three-color, fiber-coupled laser with wavelengths 488, 561, and 638 nm for fluorescence excitation. Scanning was done using a fast tip/tilt piezo mirror. For confocal detection, the emitted fluorescence was descanned, separated from the excitation by a dichroic mirror, and filtered using an emission filter (blue, 500–550 nm; green, 575–625 nm; and red, 650–750 nm). Photons were counted using fiber-coupled single-photon counting modules. The multimode fibers serve as pinholes providing background rejection. Experimental chamber: PDMS (SYLGARD 184 Silicone Elastomer) was cast on a plastic dish and cured, then cut into rectangles. PDMS rectangular walls were placed on an untreated glass coverslip (Bar Naor, Petah Tikva, Israel) and mounted onto an automated XY-stage. The GPMV sample was added to the chamber and a few drops of oil were put on the sample surface to prevent evaporation.

Micropipette aspiration

A micropipette aspiration setup including micromanipulator (Sensapex, Oulu, Finland) holding a micropipette with diameter of 5 μm (Biological Industries) connected to a Fluigent EZ-25 pump was integrated to our optical tweezer instrument. Before and after each experiment, the zero-suction pressure was found by aspirating a polystyrene bead (3.43 μm, Spherotech, Lake Forest, IL, United States) into the pipette and reducing the suction pressure until the bead stopped moving.

Tubular three-way junction formation

To pull a membrane tube, an optically trapped bead was brought in contact with the GPMV for about a minute, and then moved away from the vesicle (18).

First, two membrane tubes were pulled from aspirated GPMVs using two optically trapped beads (19). The distance between the tubes was reduced by moving one of the tubes toward the other until they coalesced and formed three membrane tube junction (19). To form swellings, the suction pressure was reduced to minimum. Then, we instantaneously increased the suction pressure to values in the range of 0.2–0.44 mbar (corresponding to 4–10 $\times$ 10^{-5} N/m membrane tension). For the confocal imaging, a 532 nm laser was used for DiI-C12 excitation with emission detected in the green channel.

Computations

The numerical minimization of the system's energy was performed using Surface Evolver (20), a program designed to find minimal energy shapes of 2D surfaces in 3D space upon satisfying boundary conditions imposed on the surface shape and constraints that can be expressed through integrals over the membrane surface. The program uses a representation of the membrane surface by the finite element method based on surface triangulation. The energy minimized by the program includes contributions related to the surface area, the volume of the system, and the mean curvature of the surface. To account for the constraints of conservation of the system's surface area and volume in the altered states, Surface Evolver uses the Lagrange method and retrieves, along with the membrane configurations, the values of the Lagrange multipliers corresponding, as mentioned in the results, to the system's tension, γ, and pressure, P, respectively.

The computational codes are publicly available at: https://github.com/benzucker-tau/three-way_bulging.

RESULTS

We experimentally explored and theoretically analyzed the effect of mechanical perturbations on the migrasome-like bulge formation in migrating cells forming extensive RFs and a biomimetic system of branched membrane tubules pulled out of GPMVs. The accessible way of perturbation was different for the two systems.

We perturbed the live-cell RFs by exposing the cells to a hypotonic medium. The idea behind this experiment was that the excess of osmotic concentration in the RF lumen generates a water influx through the RF membrane, which, in turn, increases the intraluminal pressure and, consequently, stresses the RF membranes.

In the biomimetic system, the perturbation was produced by abrupt (of a subsecond timescale) changes in the pressure of GPMV suction into the micropipette, which generated the

changes in the pulling forces applied to the tubule ends by the attached beads.

Our theoretical analysis demonstrated that, despite the difference in the type of the mechanical perturbations, the processes of membrane bulging observed in the two systems were based on a common physical background of alteration of the tubular membrane tension. Extension of the analysis revealed the universal criteria of bulging independent of a specific type of mechanical perturbation of a branched tubular membrane system.

In the following, we present the results by first formulating the common theoretical model we used for the analysis of the membrane bulging in the two experimental systems. We then describe the experimental results and their theoretical treatments separately for each system. Finally, we present a generalized theoretical treatment and the resulting universal criteria of bulging in branched tubular membrane systems.

Model

Description of the model system

We consider a membrane system consisting of three identical tubular arms emerging out of a three-way junction and symmetrically oriented with 120° angles between the arm axes (Fig. 1 *A*). The distance from the junction center to the arm end is denoted by L (Fig. 1 *A*). Each tubular arm is assumed to adopt in the vicinity of its end a circular cylindrical shape with a cross-sectional radius R (Fig. 1 *A*).

The initial state of the system is created by extracting the necessary membrane area and luminal volume out of a reservoir and shaping them into the branched tubular shape. The reservoir mimics the GPMV for the biomimetic and the cell body for the live-cell systems. The reservoir membrane is considered to be flat and subjected to a tension, γ, and, generally, a transmembrane pressure, P, equal to the difference between the pressures inside and outside of the reservoir.

In the initial state, the system is in thermodynamic equilibrium with the reservoir, which is reached due to a free exchange of the membrane area and the luminal volume between the system and the reservoir. This implies that the end of each tubular arm is subjected to a pulling force, f, that resists the draining of the system's membrane back into the reservoir and depends, therefore, on the reservoir tension, γ. The equilibration between the system and the reservoir results in certain values of the membrane area, A_0, and volume, V_0, of the system, which are set by the reservoir tension, γ, and the initial length, L_0, of the tubular arms.

We consider the initial state of the system to be subjected to either of the two alterations: a rapid increase in the system volume, V, describing the osmotic swelling of the RFs of the live cells, or an abrupt increase of the pulling force, f, as in the biomimetic system.

The goal of the theoretical treatment is to determine the system's configurations in the initial and the two altered states. By analyzing the latter, we assume that in both cases the adaptation of the system configuration to the new conditions happens much faster than the exchange of the membrane area and the luminal volume between the system and the reservoir (see below for more detail). Therefore, the altered conformations represent the internal equilibrium within the system itself but not an equilibrium between the system and the reservoir. Over longer periods, the exchange of the membrane area and luminal volume between the system and the reservoir is expected to reestablish the system-reservoir equilibrium, which is accompanied by a gradual evolution of the system configurations. Here, we consider the system's conformations existing within the time window between the relatively fast internal equilibration and the relatively slow relaxation by exchanging the membrane area and luminal volume with the reservoir.

Energy of the system

The analysis of the system configurations is based on the minimization of the system's free energy, F. Following the thermodynamic approach, we define F as a thermodynamic work of the system creation out of a flat membrane reservoir subject to a tension γ and a transmembrane pressure, P. For the initial state of the system, the reservoir is real as described in the previous subsection. For the cases of the altered states of the system, which, as explained above, are considered to be effectively disconnected from and, consequently, out of equilibrium with the *real* reservoir, the free energy, F, is related to a *virtual* reservoir which would be in equilibrium with the system if connected to it. The tension, γ, and pressure, P, of a virtual reservoir are generally different from those of the real reservoir and their formal mathematical meanings are those of Lagrange multipliers used to minimize the *internal* energy of the system under constraints of constant membrane area and luminal volume, respectively.

The first contribution to F is the bending energy of the system membrane, F_B, equal to the work of sculpting the initially flat membrane extracted from the reservoir into the curved shape of the system. We treat the bending energy, F_B, according to the Helfrich model (21). We quantify the local membrane shape at each point of the membrane surface by the total curvature, J, which is the sum of the two principal curvatures (22), and consider the area density of the bending energy, f_B, to be given by

$$f_B = \frac{\kappa}{2} J^2 \qquad (1)$$

where κ is the membrane-bending modulus (21). The total membrane-bending energy is given by

$$F_B = \int f_B dA \tag{2}$$

where the integration is performed over the entire area of the system's membrane.

By using Eq.1 we assume the membrane to have a vanishing spontaneous curvature (21), which implies that the two membrane monolayers have identical elastic properties providing the membrane with up-down symmetry. In addition, we do not consider the energy contributions related to the membrane Gaussian curvature (21) since the local membrane bulging does not affect the membrane topological genus so the Gaussian curvature energy remains constant according to the Gauss-Bonnet theorem (22).

Another contribution to the energy is the thermodynamic work of extracting the membrane area, A, out of the reservoir given by

$$F_A = \gamma A \tag{3}$$

The thermodynamic work of extracting out of the reservoir of the system's volume, V, is

$$F_V = -PV \tag{4}$$

Finally, the contribution of the work performed by a pulling force, f, applied to the end of a tubular arm and changing the tubular length L is given by,

$$F_L = -fL \tag{5}$$

It must be emphasized that, independently of whether the reservoir is real or virtual, the reservoir tension, γ, is equal to the curvature-independent contribution to the full tension in the curved membrane of the system, as defined according to Gibbs' thermodynamic approach ((23), see (24,25) for review). Keeping this in mind, we refer to γ as the system's tension, for brevity.

In the following, we use two combinations of the membrane bending rigidity, κ, tension, γ, and pressure difference, P. The first,

$$\lambda = \sqrt{\frac{\kappa}{\gamma}} \tag{6}$$

has a dimension of length and will be called below the relaxation length. The second,

$$p = P\sqrt{\frac{\kappa}{\gamma^3}} \tag{7}$$

is dimensionless and will be referred to below as the *stress parameter*.

Way of analysis

In general, the membrane configurations resulting from the minimization of the bending energy (Eqs. 1 and 2), along with the area and volume energies (Eqs. 3 and 4), can be determined by the numerical or analytical solution of the shape equations (see (24,25) for review). Yet, because of the complex shapes of the tubular junctions, solving the shape equations appears too complicated. Therefore, we performed a direct numerical minimization of the system's energy by using Brakke's "Surface Evolver" (20) (see materials and methods). The boundary conditions, which are satisfied by the Surface Evolver computations, are set on the system boundaries represented by three identical circles whose centers are located on the axes of the tubular arms at the distance, L, from the system center (Fig. 1 *A*). The plane of each boundary circle is perpendicular, while the tangent to the membrane surface is parallel to the axis of the corresponding arm (Fig. 1 *A*).

Initial state

Before analyzing the altered states of the system we computed the configuration and the energy of the system in its initial state preceding the cell exposure to a hypotonic medium or an abrupt increase of tension in the reservoir of the biomimetic system. The initial state corresponds to the equilibrium of the system with the real reservoir characterized by the tension, γ, and vanishing pressure, $P = 0$. The energy of the initial state, F_0, is a sum of the work of the membrane extraction (Eq. 3) and bending (Eq. 2),

$$F_0 = \frac{\kappa}{2}\int J^2 dA + \gamma A \tag{8}$$

The numerical minimization of the energy (Eq. 8) with respect to the membrane configuration was performed for a constant value of the arm length, L_0, without any constraints on the system membrane area, A, volume, V, and the radius of the boundary circles, R. The resulting configuration is presented in Fig. 1 *B*. The tubular arms far enough from the junction acquire the shape of a smooth circular cylinder with a cross-sectional radius proportional to the relaxation length (Eq. 6),

$$R = \frac{\lambda}{\sqrt{2}} \tag{9}$$

the relationship characterizing a single tether pulled out of a flat membrane reservoir (5). Also, the width of the tubular junction is of the order of the relaxation length, λ.

Because of the cylindrical shape of the arm ends, the pulling force applied to each of them equals (5)

$$f = 2\pi\sqrt{2\kappa\gamma} \tag{10}$$

For the following, we denote the computed initial membrane area and volume of the system by A_0, and V_0, respectively.

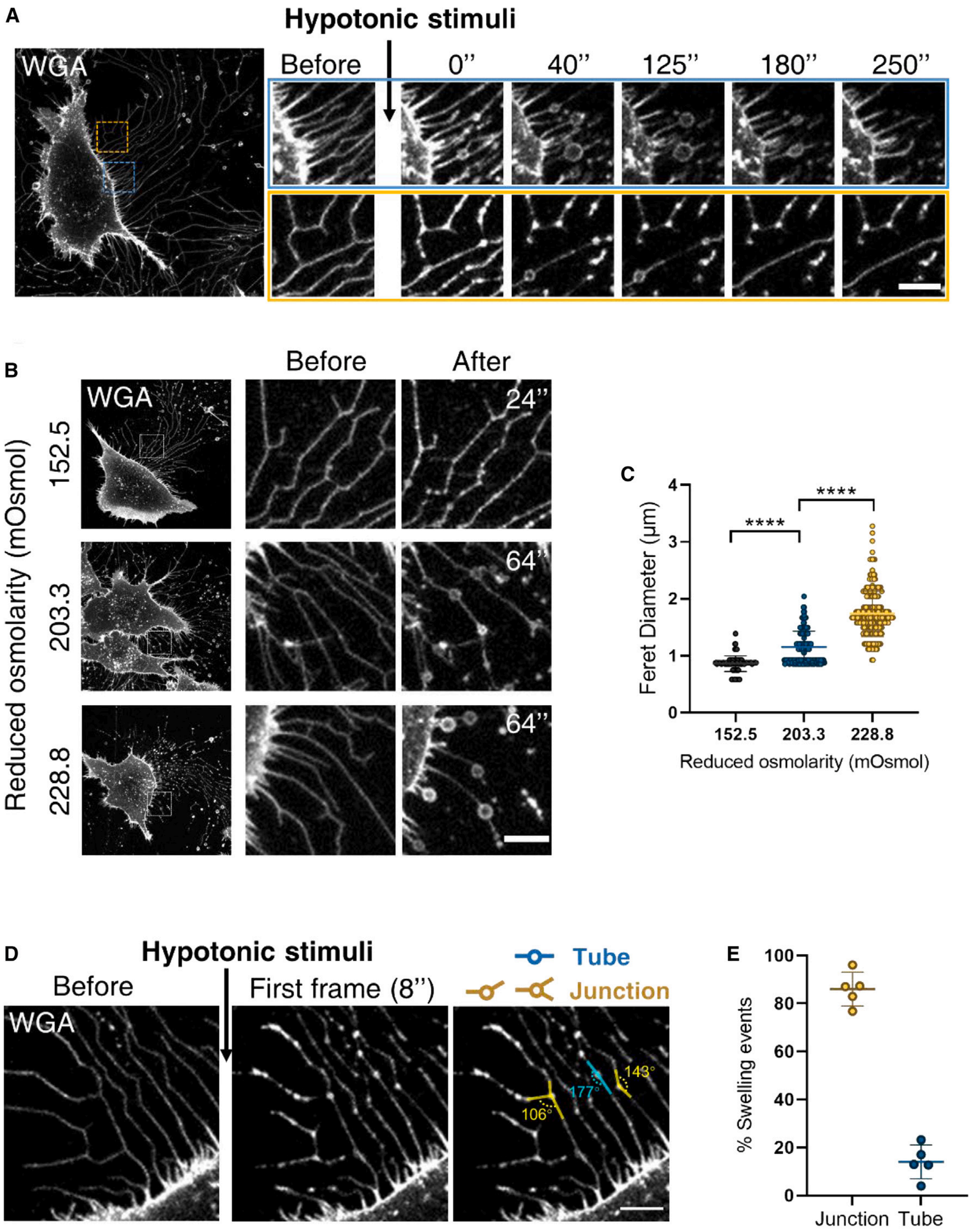

FIGURE 2 Bulge formation in retraction fibers by cell exposure to a hypotonic medium. L929 cells prestained with WGA488 in DPBS were used for all experiments in this figure. (*A*) Image series showing the progress of hypotonic-induced bulges on the retraction fibers. Cells were treated with 25% DPBS (228.8 mOsmol). Scale bar, 5 μm. (*B*) Representative confocal images of cells before and after treatment with hypotonic buffers. A 5 min 3D time-lapse series was collected to fully capture the progress of swelling. Images in the "After" panel refer to the time points when the bulges reached maximum size. The corresponding time points were marked at the right corner of the images. The reduced osmolarity labeled at the left side of each panel represents the difference between the extracellular system osmolarity before and after the hypotonic stimulation. Scale bar, 5 μm. (*C*) The size of the bulges in (*B*) were measured using ImageJ. In brief, the outer edge of each bulge was labeled using Elliptical Selection Tool and the Feret Diameter of the ellipse was calculated. Data acquired from 3 independent experiments were pooled for analysis. A total of 116, 252, or 219 bulging events were analyzed respectively. *p* values were calculated using a two-tailed unpaired nonparametric test (Mann-Whitney test). $p < 0.05$ was considered statistically significant. ****$p < 0.0001$. (*D*) Representative confocal images of a cell before (*left*) and after (*middle*) hypotonic stimulation. Cells were treated with 25% DPBS (228.8 mOsmol). The geometry

(legend continued on next page)

Bulge generation in junctions of RFs of migrating live cells by osmotic swelling

Experimental demonstration

We exposed the cells to a hypotonic solution, which stimulated the formation of local bulging on the initially smooth RFs (Fig. 2). The bulges gradually developed within a timescale of a few tens of seconds (Fig. 2 *A*). After longer periods of about 3–5 min the bulges disappeared. The typical bulge size was on a micron scale and slightly depended on the external osmolarity (Fig. 2, *B* and *C*). Importantly, more than 80% of the bulges formed on the three-way junctions (Fig. 2, *D* and *E*). Note that the junctions appeared either as regular nearly symmetric three-way vertices or as irregular vertices in which one ray was hardly recognizable, apparently, because of its shortness.

Modeling

We modeled the conformations of the live-cell RFs subject to osmotic swelling (Fig. 2). We computed the energetically favorable conformations of the system (Fig. 3 *A*) assuming the system volume, V, to increase with respect to the initial value, V_0, by an excess volume, ΔV, while the membrane area and the arm lengths remain equal to their initial values, A_0 and L_0, respectively. This implies that we address the conformations of the live-cell RFs emerging and existing within time periods that are sufficiently long for the RFs volume to substantially increase due to the osmotically driven water influx but too short for the RF membrane area to significantly change due to the exchange with the reservoir represented by the cell body. The reason for considering this regime is the hierarchy of three timescales, namely, those of RF swelling accompanied by the bulging, τ_{bulg}, the cytoplasm exchange between the RF lumen and the cell body, τ_{cyt}, and the membrane exchange between the RF and the cell plasma membrane, τ_{mem}. According to the experimental results, $\tau_{bulg} \approx 10$ s, while $\tau_{cyt} \approx 4$ min. The membrane exchange time, τ_{mem}, must be the longest one since it corresponds to the time of the tension equilibration between the RF and the plasma membrane, which is expected to be longer than 10 min for epithelial cells (26,27). Thus, the bulging is the shortest process, $\tau_{bulg} \ll \tau_{cyt}$ and $\tau_{bulg} \ll \tau_{mem}$, which justifies our consideration.

Hence, we set the system's geometrical parameters to $L = L_0$, $A = A_0$, and $V = V_0 + \Delta V$, and numerically minimize the system bending energy (Eq. 2) by using Surface Evolver (20) for constant A, V, and L, but varying radii, R, of the system's boundaries.

The computational results are presented in Fig. 3. As long as the excess volume, ΔV, remains small compared with the initial volume of the system, V_0, the system's configurations (Fig. 3 *A*) stay qualitatively similar to the initial one (Fig. 1 *B*). The tubular arms become progressively narrower while the junction inflates but its projection on the system plane retains the shape of a triangle with concave edges (Fig. 3 *A*). The extents of the tubule narrowing and the junction inflation are set by the ratio between the excess and the initial volumes, $\frac{\Delta V}{V_0}$.

When $\frac{\Delta V}{V_0}$ reaches a critical value, $\frac{\Delta V^*}{V_0}$, the character of the system evolution changes. The contour of the junction projection on the system's plane starts to lose its concavity (Fig. 3 *B*). When $\frac{\Delta V}{V_0}$ exceeds the critical value $\frac{\Delta V^*}{V_0}$, the shape of the junction projection turns into a triangle with convex edges while the bulging becomes progressively similar to a sphere (Fig. 3 *C*). We refer to the regime of the evolution of the junction's configuration corresponding to $\frac{\Delta V}{V_0} > \frac{\Delta V^*}{V_0}$ as the junction pearling.

The computed variation of the system tension γ accompanying the change of the excess volume, $\frac{\Delta V}{V_0}$, is presented in Fig. 3 *D*, which shows that the inflation and the following pearling of the junction correspond to the tension increase with a critical tension value, γ^*, determining the pearling onset. The value of the ratio between the critical and initial membrane tensions is

$$\gamma^* / \gamma_0 \approx 1.8 \tag{11}$$

Summarizing, the model predicts bulging of the system's tubular junction to be driven by a rise of the membrane tension resulting from an increase of the system volume. This explains the observed formation of the migrasome-like bulges in live-cell RFs (Fig. 2).

On longer timescales enabling the membrane exchange, the predicted membrane bulging must disappear due to the establishment of the thermodynamic equilibrium between the RFs and the cell plasma membrane, as experimentally observed. The explicit analysis of this stage of the system evolution is beyond the present analysis.

Bulge generation in junctions of biomimetic membrane tubules by abrupt increase of tension

Experimental demonstration

As a biomimetic setup imitating branched RFs, we used three membrane nanotubules connected by a three-way junction (Fig. 4 *A*). To create this system, GPMVs were generated from HEK293T cells (see materials and

of the sites of bulging events was identified by measuring the angles between connecting retraction fibers. Bulging sites with angles $\geq 165°$ were identified as "Tube", whereas sites with angles $<165°$ were identified as "Junction". Two representative measurements of junctions and one representative measurement of a tube are labeled in yellow and blue, respectively (*right*). Scale bar, 5 μm. (*E*) Statistical analysis of the site of swellings in (*D*). Data acquired from 3 independent experiments were pooled for analysis. A total of 273 swelling events in 5 cells were analyzed.

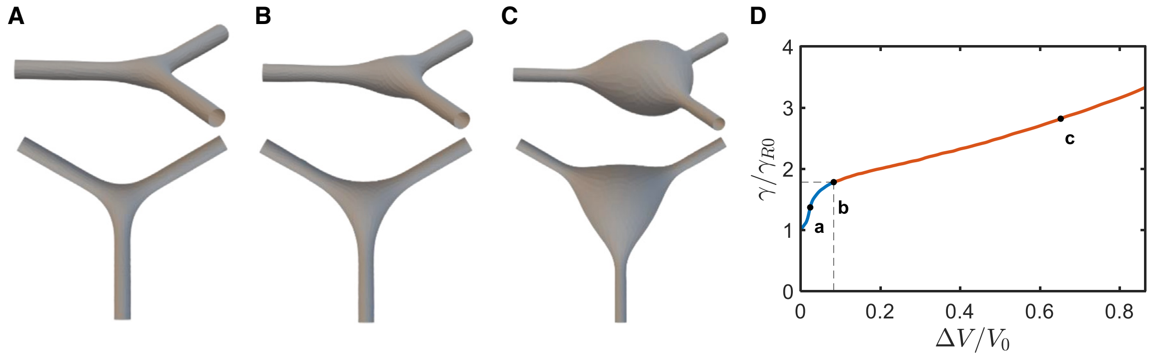

FIGURE 3 Modeling the junction pearling by increased tension resulting from an increase of the luminal volume. (*A–C*) Simulations of a junction connecting three tubules with a fixed membrane area and increasing volumes. (*A*) The small relative excess volume $\Delta V/V_0 = 0.024$ results in a modest expansion of the junction (*top panel*), while the contours of the junction sides remain concave (*bottom panel*). (*B*) The onset of the junction pearling transition for the critical value of the relative excess volume of $\Delta V^*/V_0 = 0.083$. (*C*) The relative excess volume $\Delta V/V_0 = 0.652$ exceeding the critical value results in a substantial bulging of the junction. The contours of the junction sides are convex (*bottom panel*) while the overall shape is more spherical (*top panel*). (*D*) The system tension as a function of the normalized volume increment. The blue (*a*) and red (*c*) segments describe, respectively, the pre- and postbulging regimes. The point (*b*) and the dashed lines indicate the pearling transition. The initial geometrical parameters of the system are $V = 111\ \lambda^3$ and $L = 70\ \lambda$ where $\lambda = \sqrt{\kappa/\gamma_R}$, and γ_R is the is the reservoir tension. The sizes chosen to agree with initial radius of $R_0 = 50$ nm and a total tubular length of $3L_0 = 5\ \mu$m. (*A–C*) Show the junction and the tubular arms up to a distance of $10 \times \lambda$ from the center of the junction.

methods). Next, the vesicles were added to a custom-made chamber mounted on the stage of a correlated optical tweezers confocal fluorescence microscope. A micropipette aspiration setup was incorporated into the tweezers-confocal system. The GPMV membrane was subjected to tension by micropipette aspiration such that the tension values were determined by the controlled suction pressure (see materials and methods). Two optically trapped polystyrene beads were pushed toward the aspirated GPMV and two membrane nanotubes were pulled out of the vesicle (Fig. 4 *A*). The distance between the tubules was reduced by moving one of them toward the other until the tubules coalesced and formed a three-way junction (Fig. 4 *A*) (19).

After the junction formation, the suction pressure was reduced to zero (Fig. 4 *B*, t = 10 s). Then the suction pressure was instantaneously increased to values in the range of 0.2–0.44 mbar corresponding to the GPMV membrane tension in the range of 0.04–0.1 mN/m. The tension increase applied by changing the suction pressure was accompanied by a step-like change in the forces applied to the beads at the tubule ends (Fig. 4 *C*).

This resulted in the formation of membrane bulges reminiscent of migrasomes (Fig. 4 *B*; Video S1). The bulges appeared in the junction and the tubules practically immediately (within subseconds) after the abrupt increase in the membrane tension (Fig. 4 *B*, t = 16 s). Yet, the tubule bulges were less stable. They shrunk and moved toward the junction (Fig. 2 *B*, t = 33 s) and, finally, merged with the junctional bulge (Fig. 4 *B*, t = 49 s).

The results of 16 realizations of this experiment are presented in the supporting material (Fig. S1).

Modeling

We modeled the configurations of the biomimetic system resulting from an abrupt increase of the tension in the GPMV and the related abrupt increase of the pulling force, f, applied to the ends of the tubular arms by the attached beads (Fig. 4). For this purpose, we determine the conformations of the computational system (Fig. 1 *A*) resulting from an instantaneous rise of the pulling force, f, compared with its initial value, f_0. In this computation, we kept the membrane area and the volume of the system equal to their initial values, A_0 and V_0, while the length, L, and the radius, R, of the tubular arm ends were free to change during the energy minimization. These assumptions are justified by the essence of the experiment in the biomimetic system. In these experiments, the tension in the reservoir (GPMV) was increased by a step-like (of a subsecond timescale) jump of the suction pressure in the micropipette. This tension increase must practically instantaneously spread all over the 10μm large tubular system since in membranes devoid of cytoskeleton the tension is expected to propagate with the speed of sound of 0.1–1 cm/s (28), so the propagation time must be less than 0.1 ms, which is orders of magnitude smaller than all the other relevant timescales (see below). The pulling forces are set by the system's tension and, thus, instantaneously follow the changes in the reservoir (GPMV) tension. Furthermore, the instantaneous propagation of tension implies that, at every time point, the tension in the system remains in equilibrium with that in the real reservoir so that there is no driving force for changes in the system's area, which justifies the assumption of constant area, A_0. The assumption of constant volume, V_0, is justified by the experimentally observed shortness of

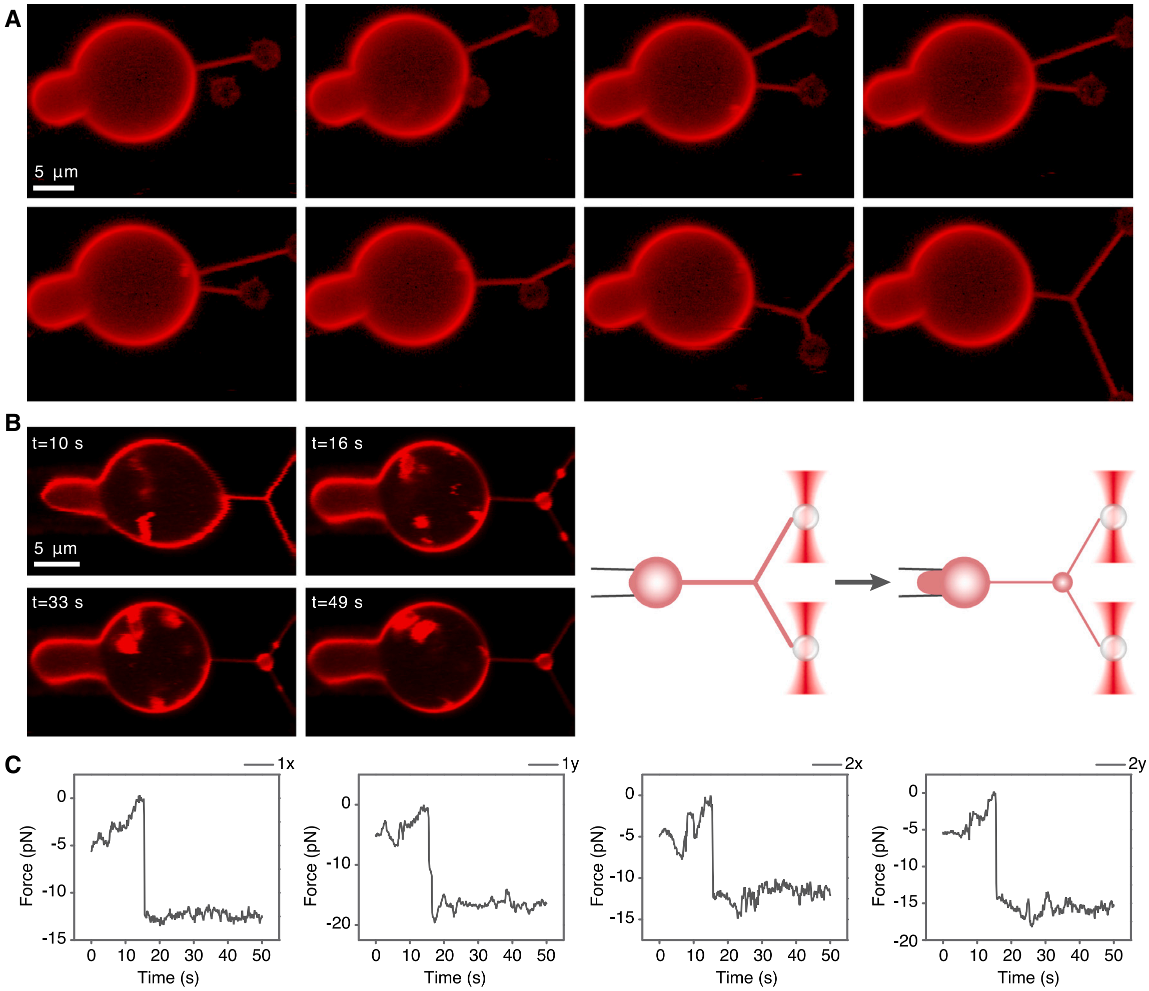

FIGURE 4 Bulge formation by abrupt (subsecond long) tension increase in the biomimetic system. (*A*) Three-tube junction formation. Confocal microscopy images of three-tube junction formation from an aspirated GPMV dyed with DiI-C12. Two membrane tubes were pulled from the vesicle. Next, the upper tube was elongated and brought toward the other tube until the tubes coalesced. The fluorescence intensity of the images is presented in log scale. (*B*) Bulge formation by tension jumps. Time-lapse confocal microscopy images of the tension jump assay. First, the suction pressure was reduced to zero (t = 10 s), and then the suction pressure was increased instantaneously to 0.39 mbar (t = 16 s), corresponding to 0.074 mN/m membrane tension, leading to a bulge formation. The small bulges that formed in the tubules moved toward (t = 33 s) and merged with the junctional bulge (t = 49 s). A video of this process can be seen in (Video S1). This experiment was performed 17 times on 11 different GPMVs in 5 independent experiments. A schematic illustration is shown on the right. (*C*) Force plots of the tension jump presented in (*B*). The left panels correspond to the x and y force components measured from the bead1 displacement (from optical trap1) and the right panels correspond to the x and y force components measured from the bead2 displacement (from optical trap2).

the time of bulging estimated to be in the subsecond range $\tau_{bulg} \ll 1$ s compared with the time, τ_{vol}, of the volume exchange between the system and the reservoir accompanying the relaxation of the system and disappearance of the bulges. Indeed, an empirical consideration provides a low-limit estimation of τ_{vol} to be of the order of seconds, while the observed relaxation time was of the scale of minutes (see, for example, Video S1). The slowness of the observed relaxation was, most probably, related to the effect on the hydrodynamic resistance of the GPMV membrane tubules produced by proteins connected to the luminal membrane face.

According to this scenario, we used Surface Evolver (20) to compute the configurations adopted by the system as a result of an instantaneous increase of the pulling forces by minimizing the sum of the bending energy (Eqs. 1 and 2) and the work of pulling the three arms (Eq. 4),

$$F = \frac{\kappa}{2}\int J^2 dA - 3fL, \tag{12}$$

upon the constraints of constant area, A_0, and volume, V_0, but allowing variations of the arm length, L, and the radius of the system's boundary circles, R.

The computational results presented in Fig. 5 demonstrate that, as long as the pulling force, f, only slightly deviates from its initial values, f_0, the system's conformation undergoes relatively small changes consisting in a moderate redistribution of the volume between the tubules and the junction while the latter retains qualitatively similar shapes

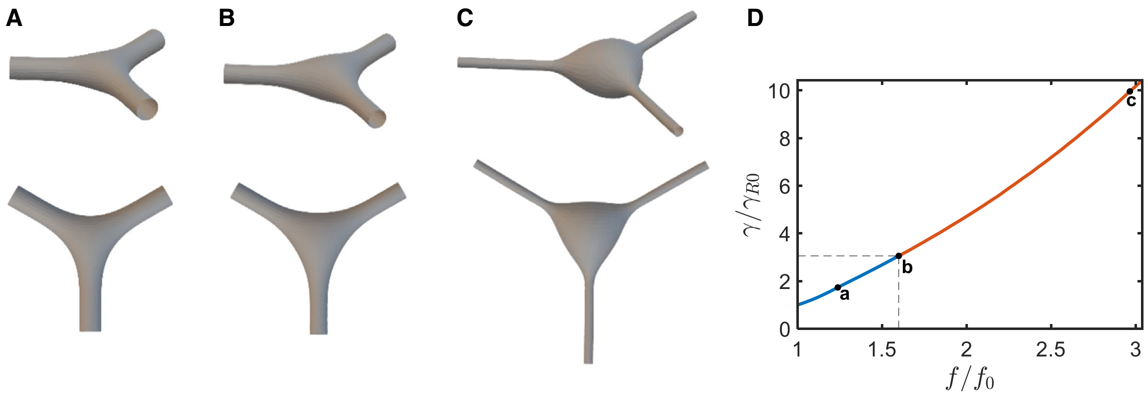

FIGURE 5 Modeling the junction pearling by increased tension due to abrupt application of elevated pulling force. (*A*–*C*) Computational determination of the energetically favorable configurations of the junction system. The arm ends are subjected to equal pulling forces. The simulations were performed under conditions of fixed overall membrane area and volume of the system and increasing value of the pulling forces. In the initial state, the pulling force value is f_0. (*A*) A small increment of the relative pulling force, $f/f_0 = 1.2$, results in a modest expansion of the junction. (*B*) A relative pulling force $f/f_0 = 1.5$ close to the critical value, $f^*/f_0 \approx 1.6$. (*C*) A pulling force of $f/f_0 = 2.8$ exceeding the critical value results in a substantial bulging of the junction. (*D*) The dependence of tension on the normalized pulling force. The points on the curve correspond to the three simulations shown in (*A*–*C*). The blue (*a*) and red (*c*) segments describe, respectively, the pre- and postbulging regimes. The point (*b*) and the dashed lines indicate the pearling transition. The initial spatial parameters of the system are $V = 28\,\lambda_0^3$ and $L = 5.3\,\lambda_0$ where $\lambda_0 = \sqrt{\kappa/\gamma_{R0}}$, and the initial tension is γ_{R0}.

of triangles with concave edges (Fig. 5 *A*). Application of a pulling force larger than the critical values, f^*, leads to pearling of the junction (Fig. 5 *C*). The system's membrane tension, γ, is related to the pulling force, f, as presented in Fig. 5 *D*. Hence, the onset of the junction pearling is determined by the critical membrane tension, γ^*. The computed ratio between the critical and the initial tensions is

$$\gamma^*/\gamma_0 \approx 3 \tag{13}$$

Summarizing these results predicts the formation of the migrasome-like bulge in the tubular junctions as observed in the biomimetic system (Fig. 4) upon an abrupt increase of the reservoir tension and the related pulling forces.

Common criterion of the junction bulging for the experimentally explored alterations of the system

In the previous sections, we found that the bulging occurs at a critical membrane tension, γ^*, which corresponds to the critical value of the relative excess volume, $\frac{\Delta V^*}{V_0}$, or of the ratio between the increased and the initial pulling force, $\frac{f^*}{f_0}$, depending on the mode of the system alteration used in our experiments. For both alterations, the bulging was predicted to occur primarily in the junction rather than in the tubular arms of the system. Yet, the numerical value of the critical tension, γ^*, was found to be different for the two alterations.

Here, we demonstrate the existence of a common criterion for the pearling onset in the two considered cases. For this purpose, we computed the variation of the stress parameter p (Eq. 7) accompanying the changes in the relative excess volume, $\frac{\Delta V}{V_0}$, for the first, and of the relative pulling force, $\frac{f}{f_0}$, for the second alteration. The results are presented in Fig. 6, *A* and *B*, respectively.

According to these results, the described above criteria of the junction pearling transition for the two alterations, namely, the critical relative excess volume, $\frac{\Delta V^*}{V_0}$, and the critical relative pulling force, $\frac{f^*}{f_0}$, correspond to the same critical value of the stress parameter,

$$p^* \approx 0.4 \tag{14}$$

Hence, the critical value of the stress parameter (Eq. 14) represents the common criterion of the junction pearling for the two considered alterations of the system.

Comparison of the bulging criteria for a tubular junction and a cylindrical tubule

It is instructive to compare the obtained criterion of junction bulging (Eq. 14) with that of bulging of a cylindrical tubule. The latter criterion can be analytically obtained by using the membrane shape equation (24, 25). In the particular case of a cylindrical membrane the, generally, complex and nonlinear shape equation acquires a simple form,

$$P - \gamma J + \frac{1}{2}\kappa J^3 = 0 \tag{15}$$

where the curvature J is related to the radius of the cylinder, R, by $J = \frac{1}{R}$.

The values of the tension, γ, and pressure, P, for which (Eq. 15) has no positive solution for the curvature, J, correspond to a loss of stability of a cylindrical shape. A simple analysis of Eq. 15 shows that the instability onset is determined by the critical value of the stress parameter

$$p^*_{cyl} = \left(\frac{2}{3}\right)^{3/2} \approx 0.54 \tag{16}$$

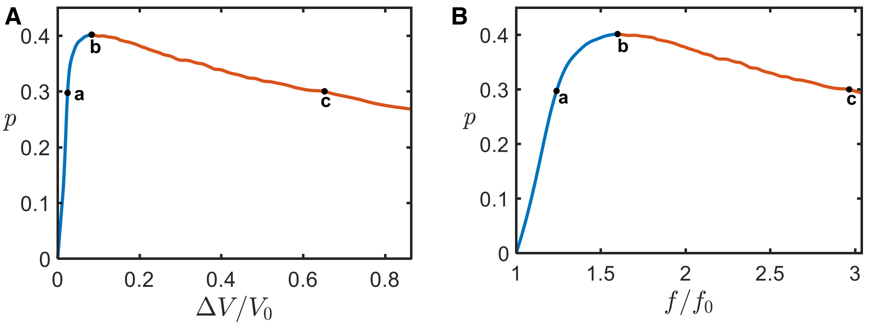

FIGURE 6 The evolution of the stress parameter under the two perturbations of the system considered in the main part. (*A*) The dependence of the stress parameter on the relative volume addition corresponding to (Fig. 3). The blue (*a*) and red (*c*) segments describe, respectively, the pre- and postbulging regimes. The maximal value of the stress parameter p indicated by "b" represents the critical value of the stress parameter, $p^* \cong 0.4$, setting the junction pearling transition. (*B*) The stress parameter as a function of the normalized force corresponding to (Fig. 5). The maximal value of the stress parameter p indicated by "b" represents the critical value of the stress parameter, $p^* \cong 0.4$, setting the junction pearling transition.

which is larger than that of a tubular junction (Eq. 14). To confirm this conclusion, we performed Surface Evolver computations of the energetically favorable conformations of an axially symmetric shape for a given length, L, pressure, P, and tension, γ. These computations confirmed that, as long as the stress parameter is smaller than the critical value given by Eq. 16, $p < p^*_{cyl}$, the system acquires an equilibrium configuration of a smooth cylinder whose radius depends on p. If the stress parameter exceeds the critical value $p > p^*_{cyl}$, no equilibrium configuration could be computationally found.

Thus, tubular junctions are more susceptible than tubules to the pearling transition, which implies that in our system consisting of a tubular junction and three tubular arms bulging of the junction is more favorable than that of the arms. This explains our observations in the biomimetic system according to which the bulges emerging on the tubular arms concomitantly with the bulging of the junction exhibit instability and disintegrate by shrinking and moving toward and eventually merging with the junctional bulge (Fig. 4). In the supporting material, we present the results of an explicit computational analysis demonstrating that the state of the system in which the bulging occurs only in the junction is favorable compared with the states of coexistence between the junctional and the tubular arm bulges (Fig. S2).

Universal features of mechanically altered membrane tubules and tubular junctions

Here, we generalize the results of the previous subsection and computationally analyze the configurations and the bulging transition in a cylindrical tubule and a three-way tubular junction resulting from all possible alterations on the initial smooth state. We show that for each of the two systems the parameters of the energetically favorable configurations resulting from different alterations of the initial state and different conditions of the system's adaptation to the alterations are described by universal "master" relationships between the thermodynamic variables. These computationally obtained master relationships enable one to recover the parameters of all the configurations for any alteration and conditions of adaptation. One consequence of this universality is that, independently of the kind of alteration, the bulging onset is determined by the same value of the stress parameter equal to $p^* \approx 0.4$ (Eq. 14) and $p^* \approx 0.54$ (Eq. 16) for, respectively, a three-way junction and a cylindrical tubule.

We start with considering the membrane of cylindrical topology to introduce most intuitively the major notions and the way of calculations. Then we use the same approach for analysis of the system consisting of three tubular arms connected by a symmetric three-way junction. The two systems will be referred for brevity to as, respectively, the cylinder and the junction. To facilitate the reading we repeat some of the definitions already introduced in the previous sections.

Cylinder

We consider an axially symmetric membrane of the cylindrical topology (Fig. 7 *A*). As the extensive thermodynamic variables of the membrane system, we consider its geometrical dimensions: the length along the symmetry axis, L, the surface area, A, and the luminal volume, V. As the intensive thermodynamic variables conjugated to L, A, and V, we consider, respectively, the value, f, of the pulling forces applied to the system's ends, the membrane tension, γ, and the transmembrane pressure difference, P.

As already defined above, by the tension, γ, we mean here the curvature-independent part of the full Gibbs tension (23, 25) that is equivalent to the tension in a flat membrane reservoir equilibrated with the cylindrical membrane.

Our goal here is to analyze the energetically favorable configurations of the system within different thermodynamic ensembles. Each ensemble is characterized by the set of the thermodynamic variables that can be varied for the energy minimization. For example, consideration of the ensemble (γ, V, L) means that γ, V, and L must be kept constant during the energy minimization, while the area, A, pressure, P, and pulling force, f, can be varied. To calculate the energy for each ensemble, we use the above-introduced definitions of the energy contributions (Eqs. 1, 2, 3, 4, and 5).

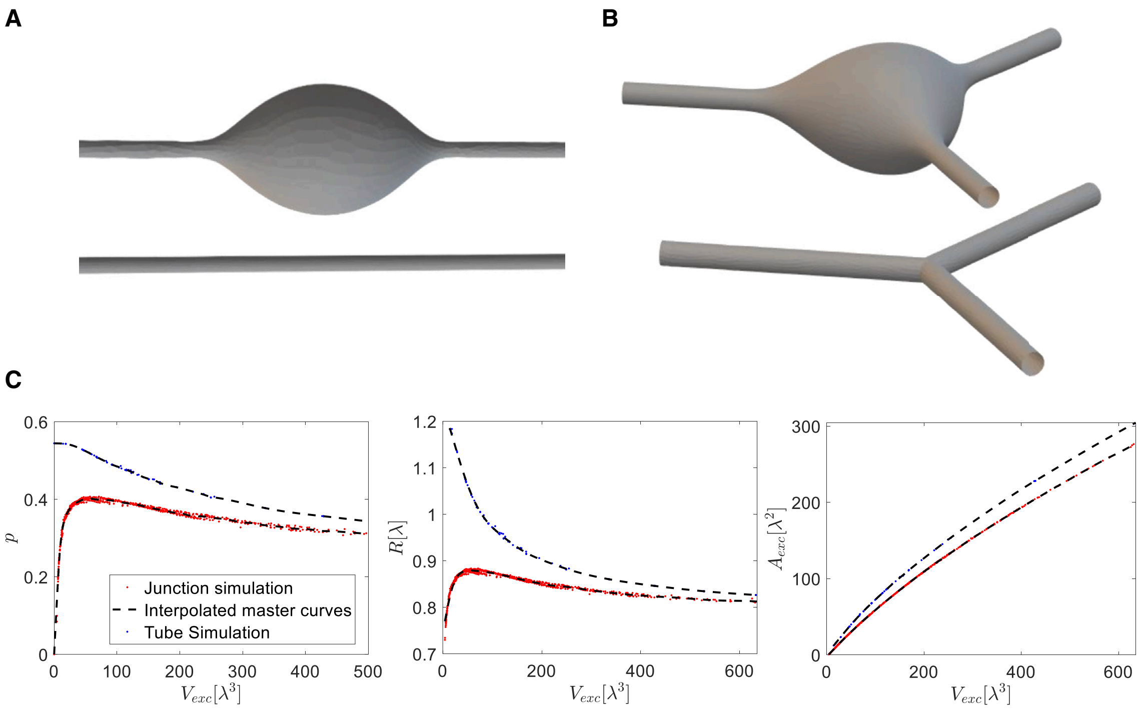

FIGURE 7 Computational results for the energetically favorable states. (*A*) An example of a computed confirmation of bulge in one-cylinder system (*top panel*) and the corresponding conformation-for-comparison (*bottom panel*), which has similar length, tension, and pressure as the resulting shape of the top panel. (*B*) An example of a computed conformation of bulge in junction (*top panel*) and its conformation-for-comparison (*bottom panel*). (*C*) The master curves. Left: the stress parameter, *p*, of tubules and junctions as a function of the excess volume. Middle: the normalized radius of the tubular ends as a function of the excess volume. Right: the excess area as a function of the excess volume. The blue and red dots represent the computational result for the cylinder and the junction system, respectively. The dashed curves are interpolations of these data.

We use the following procedure to compute the energetically favorable system configurations.

First, we chose the initial state of the system. We define this state as that of the tension, γ_0, the vanishing pressure, $P_0 = 0$, and the length, L_0. It can be easily shown using the membrane shape equation (Eq. 15) that, in the initial state, the membrane has a shape of a homogeneous cylinder with radius (21)

$$R_0 = \sqrt{\frac{\kappa}{2\gamma_0}} \tag{17}$$

We take the initial length to be considerably larger than the initial radius, $L_0 \gg R_0$. The system's initial area and volume are $A_0 = 2\pi R_0 L_0$ and $V_0 = \pi R_0^2 L_0$.

Next, we computationally look for the energetically favorable configurations acquired by altered systems in which some of its thermodynamic variables are changed with respect to their initial values and then kept constant, while the others can vary under the constraints of a certain ensemble. For example, we consider the system to be altered by the addition of an extra volume, ΔV, after which the system evolutes to an energetically favorable configuration by keeping constant the tension, $\gamma = \gamma_0$, the volume, $V = V_0 + \Delta V$, and the length, $L = L_0$, while varying the system area, A, the pressure, P, and the pulling force, f. This means that we computationally look for the energetically favorable configuration of the system within the ensemble (γ, V, L). In a separate computation, we determine the energetically favorable configuration within the ensemble (A, V, f) by keeping constant the area, $A = A_0$, the volume, $V = V_0 + \Delta V$, and the pulling force, $f = f_0$, while varying the membrane tension, γ, the pressure, P, and the length, L. Ultimately, we determine the energetically favorable configurations in all ensembles of constant volume $V = V_0 + \Delta V$. Analogously, we computationally analyze the results of the system alterations by increasing its membrane area to $A = A_0 + \Delta A$, changing the pulling force, $f = f_0 + \Delta f$, pressure, $P = P_0 + \Delta P$, and tension, $\gamma = \gamma_0 + \Delta\gamma$. For each alteration we obtain the results for all ensembles compatible with the alteration. In this way, we computationally analyze the results of all possible alterations of the systems under all possible constraints.

The energy minimization and computations of the corresponding membrane configurations were performed using Surface Evolver (20). As already mentioned, the minimized energy included the bending energy, F_B (Eqs. 1 and 2), and the energy contributions related to the changes of those extensive variables whose variations were allowed by the

ensemble (Eqs. 3, 4, and 5). At the same time, for each specific ensemble the energy minimization procedure kept constant the required variables.

For example, if the energetically favorable configuration resulting from the system alteration by a volume addition was determined within the ensemble (γ, V, L), the minimized energy consisted of the bending contribution and that of the work of changing the membrane area, $F = F_B + \gamma A$, and the minimization procedure was conducted upon the constraints of constant volume, V, and length, L. Analogously we computed the energetically favorable conformations and their energies for all possible system alterations within all the ensembles compatible with each alteration.

In practical terms, in all cases, Surface Evolver minimized the function

$$\overline{F} = F_B + \gamma A - \gamma V - fL \tag{18}$$

Depending on the ensemble, for each pair of the conjugated thermodynamic variables one was held constant while the other could change during the minimization of $\overline{F}$.

The computations in all ensembles except those keeping constant tension and pressure provided two types of the system's shapes: smooth circular cylinders and cylinders with a bulge in the middle and smooth cylindrical ends (Fig. 7 *A*, *top*). For the ensembles of constant tension and pressure, (γ, P, L) and (γ, P, f) instead of a bulged cylinder our computations exhibited infinitely inflating shapes. In the following we will discuss only the ensembles for which either the area or the volume or both of them are kept constant.

To present the quantitative characteristics of the membrane conformations resulting from our computations we introduce several notions. First, we define the conformation-for-comparison, which is a homogeneous circular cylinder whose cross-sectional radius R is equal to that of the ends of computed membrane conformation and the length is equal to that of the system, L (Fig. 7 *A*, *bottom*). If the computed shape of the system is a smooth cylinder, the conformation-for-comparison just coincides with that of the system. If the computed shape has a bulge, the conformation-for-comparison deviates from the computed one underneath the bulge. Obviously, the volume, $V_C = \pi R^2 L$, and area, $A_C = 2\pi RL$, of the conformation-for-comparison deviate from those of the computed conformation, V and A, unless the latter is a smooth cylinder.

Next, we define the dimensionless values referred below to as the excess volume, V_{exc}, and the excess area, A_{exc}, of the system. The excess volume is the difference between the real volume and that of the conformation-for-comparison normalized by the third power of the relaxation length λ (Eq. 6),

$$V_{exc} = \frac{V - V_C}{\lambda^3} \tag{19}$$

The excess area is defined analogously,

$$A_{exc} = \frac{A - A_C}{\lambda^2} \tag{20}$$

The results of our numerical computations of the energetically favorable system conformations can be described by the stress parameter, p (Eq. 7), the excess volume, V_{exc} (Eq. 19), excess area A_{exc} (Eq. 20), and the normalized radius of the system's boundary, $\frac{R}{\lambda}$, characterizing each conformation. The computationally obtained relationships between these parameters are presented in Fig. 7 *C* by blue dots which include the results for all possible kinds of alterations and within all ensembles compatible with each alteration. As demonstrated by Fig. 7 *C*, all the results, independently of the kind of the system's alteration and the ensemble type, converge to common universal curves referred to below as the master curves.

The most instructive among the master curves is that relating the stress parameter, p, and the excess volume, V_{exc} (Fig. 7 *C*, *left panel*, *blue dots*). It must be noted that, besides the well-visible points of this curve corresponding to $V_{exc} > 0$, there are points located on the ordinate axis and describing the situations in which the system has smooth cylindrical configuration coinciding with the conformation-for-comparisons and therefore having vanishing excess volume, $V_{exc} = 0$, and area, $A_{exc} = 0$, for nonvanishing parameter, $p \neq 0$. According to the master curve (Fig. 7 *C*, *left panel*, *blue dots*), the critical value of the stress parameter p determining the onset of bulging, $V_{exc} > 0$, equals $p^* \approx 0.54$ for all alterations and ensembles of the system.

The master curves (Fig. 7 *C*, *blue dots*) along with the characteristics of the initial state and the specific alteration enable recovering of all the thermodynamic parameters of every computationally obtained conformation of the system.

For example, we consider again as the initial state a smooth cylinder subjected to tension, γ_0, a vanishing pressure, $P_0 = 0$, and characterized by the length, L_0, the cross-sectional radius, R_0, given by Eq. 17, and the corresponding area, A_0, and volume, V_0. The alteration is the addition of extra volume, ΔV. We consider the system to be described by the ensemble (A, V, L) and seek to determine the tension, γ, the pressure, P, and the pulling force, f, of the energetically favorable conformation using the master curve (Fig. 7 *C*, *blue dots*). This can be done by solving the following system of equations:

$$V_0 + \Delta V = V_{exc}\left(\frac{\kappa}{\gamma}\right)^{\frac{3}{2}} + \pi L_0 R^2 \tag{21}$$

$$A_0 = A_{exc}\frac{\kappa}{\gamma} + 2\pi L_0 R \tag{22}$$

$$P\sqrt{\frac{\kappa}{\gamma^3}} = f_p(V_{exc}) \tag{23}$$

$$A_{exc} = f_A(V_{exc}) \tag{24}$$

$$R = f_R(V_{exc}) \tag{25}$$

Equations 21 and 22 represent the conditions of the constant volume and area, respectively. The functions $f_p(V_{exc})$, $f_A(V_{exc}), f_R(V_{exc})$, in Eqs. 23, 24, and 25, represent the master curves. Solving the five equations (Eqs. 21, 22, 23, 24, and 25) with respect to five unknowns, P, γ, R, V_{exc}, A_{exc}, gives the pressure, tension, and radius of the configurations as the functions of the initial conditions and the alteration $P(V_0, A_0, \Delta V)$, $\gamma(V_0, A_0, \Delta V)$, $R(V_0, A_0, \Delta V)$. The pulling force can be found according to $f = 2\pi R\gamma - \pi R^2 P$.

Junction

Here, we consider the junction system consisting of three cylindrical tubules connected by a three-way intersection, as described above (Fig. 7 *B*, *top*). We perform the computational analysis according to the same approach and using the same type of definitions as introduced above for the cylinder.

The only new issue is the conformation-for-comparison of the junction system. We choose it to consist of three cylinders each having the cross-sectional radius, R, equal to that of the cylindrical endings of a tubular arm, and the length equal to that of the tubular arm, L, i.e., the distance between the arm's end and the center of the junction (Fig. 7 *B*, *bottom*). The excess volume, V_{exc}, and area, A_{exc}, of the system are defined according to Eqs. 19 and 20, where V_C and A_C are the volume and area of the conformation-for-comparison.

As above, we consider the initial state of the junction system to be characterized by the tension γ_0, the vanishing pressure, $P_0 = 0$, the radius R_0 of the arm ends related to γ_0 by Eq. 17, and the arm length, L_0, substantially exceeding the radius, $L_0 \gg R_0$.

In contrast to the cylinder considered above, the excess values, V_{exc}, A_{exc}, of the junction system never vanish, not even in the initial state, because of the noncylindrical geometry of the junction core.

Our computations showed that all kinds of alterations of the junction system can drive bulging if exceeding some critical value and the bulging always develops in the junction itself rather than in a tubular arm (see a substantiation of this finding in the supporting material).

In full analogy to the cylinder system, the parameters of the minimal energy conformations of the junction system obtained for all alterations of the initial state and for all ensembles compatible with these alterations converge to the master curves, which are presented in (Fig. 7 *C, red dots*). On the master curve relating the stress parameter, p, and the excess volume, V_{exc} (Fig. 7 *C*, *left panel*, *red dots*), the parameters of the configurations corresponding to the bulged junction are represented by the points on the right-hand side of the maximum. This master curve shows that, also for the junction system, the bulging transition for all alteration and ensembles is determined by a common critical value of the stress parameter $p^* \approx 0.4$.

Finally, also for the junction system the parameters of the minimal energy configurations for each alteration and all the compatible ensembles can be found using the master curves and the conditions accounting for the conserved values, as described by Eqs. 21, 22, 23, 24, and 25.

It has to be emphasized that this universal description of the system is valid only if the tubular arms are sufficiently long compared with the dimension of the junction.

DISCUSSION

Migrasomes are micron-scale vesicle-like organelles emerging on cell RFs during cell crawling on external substrates (1). The preferred location of migrasomes is the sites of three-way junctions between the tubular regions of branched RFs (1). The migrasome formation was shown to occur in two steps: the first step of the initial local RF swelling into micron-large spherical bulges, and the second step of the bulge maturation and stabilization by TSPAN proteins. While the physical factors underlying the second step were studied (12,14), the forces responsible for the first step have not been addressed.

Here, we propose that the first step of migrasome formation can be mediated by the increase of the membrane tension, the major mechanical factor driving the formation of the RFs themselves and also controlling multiple other cellular processes, which involve membrane shaping and remodeling.

We substantiated this proposal experimentally by using live cells and a biomimetic system, and by computationally analyzing the energetically favorable membrane configurations. To increase the membrane tension in the live-cell system we used the cell swelling in a hypoosmotic medium. We exposed live cells lacking TSPANs and having well-developed systems of RFs to solutions of low osmotic concentrations and observed the transient formation of membrane bulges preferentially in the junction sites of RF tubular branches. In the biomimetic system consisting of three membrane tubules connected by a tubular junction and pulled out of a GPMV, we changed the tension by an abrupt increase of the suction pressure applied to the GPMV and the related sharp increase of the forces pulling the ends of the tubules. This initiated the formation of migrasome-like bulges with a preferred location in the junction site. Finally, we theoretically analyzed the energetically favorable configurations adopted by a system of three membrane tubules connected by a three-way junction upon an increase of the membrane tension by either an increase of the system luminal volume or an abrupt increase of the pulling forces. We also derived a universal criterion determining the onset of the system bulging independently of the specific way of

increasing the tension. The computational analysis predicted the preferential bulge formation in the junction compared with the tubular arms. Altogether, we present compelling evidence of the membrane tension being the force able to initiate the migrasome formation in the three-way junctions of branched RFs.

From the perspective of membrane physics, the formation of membrane bulges referred to in the literature as the pearling transition or peristaltic instability has been extensively studied for cylindrical membrane tubules and appears to be a rather ubiquitous phenomenon typical for both lipid vesicles and cell membranes (29,30). Phenomenologically, this transition involves the loss of stability in initially smooth tubular shapes, which leads to the formation of chains of bead-like membrane bulges connected by cylindrical segments. These configurations appear qualitatively similar across membrane tubules from different and unrelated origins. Yet, despite the resemblance of the membrane configurations, the physical factors driving the transitions vary for different systems. The pearling transition has been attributed to factors such as longitudinal stretching of tubule-like vesicles upon conservation of the tubule volume (31), osmotic swelling of axons by dilution of the external medium (32), generation of a large intrinsic curvature of membranes by membrane-bound or inserted molecules (30,33), and depolymerization of intracellular actin (29,34). The pearling transition resulting from the vesicle stretching was proposed to be analogous to tension-driven Rayleigh instability opposed by the membrane resistance to bending rigidity (31), hydrodynamic drag (35), rigidity of the submembrane actin shell (29), gel-like dynamics of the cytoskeleton, and the membrane-cytoskeleton coupling (32).

The phenomena studied here can be considered as a generalization of the pearling transition to systems of branched membrane tubules containing junctions. The essence of our finding is that tubular junctions are more susceptible to the pearling transition than tubules. We predicted the transition in the junction to occur at lower pressure/tension ratios. This conclusion explained our observations of the generation of the migrasome-like bulges, preferably in the junctions of the RFs, and of the tendency of the bulges formed on the tubular arms of the biomimetic system to merge with the junctional bulge.

It is important to emphasize that, in cell membranes, the purely physical picture described and analyzed here must be modulated by specific biological factors. A recent work demonstrated an essential role of PIP5K1A, a PI4P kinase that converts PI4P into PI(4,5)P_2 (36), and of sphingomyelin synthase 2 (SMS2) in migrasome biogenesis (37). Foci of SMS2 protein initially formed at the cell leading edge moved to the RFs during the cell migration and served as the sites of migrasome nucleation. According to the proposed model, in the case where an SMS2 foci is formed within a tubular region of a RF the resulting nascent migrasome must move into the nearest junction. Another possibility is that such a nucleation site has a lower chance of developing into a mature migrasome compared with an SMS2 foci located at three-way junctions.

A possible biological rationale for migrasome formation in the junctions rather than the linear tubular regions of branched RFs is kinetic facilitation of the migrasome loading with luminal and membrane cargos.

Finally, it must be mentioned that the mechanically driven mechanism of the migrasome initiation considered here is, most probably, not the only one acting in membranes of live cells. Other types of mechanisms based on redistribution into the RF junctions of specific lipids modifying the membrane's elastic properties and of proteins other than TSPANs cannot be excluded. Such mechanisms are expected to be synergistic with the considered here mechanism fueled by mechanical forces.

ACKNOWLEDGMENTS

M.M.K. was supported by the Israel Science Foundation (grant no. 1994/22) and holds Joseph Klafter Chair in Biophysics. Raya Sorkin acknowledges support by the Israel Science Foundation (grant no. 1289/20) and the NSF-BSF (grant no. 2021793), and holds the Raymond and Beverly Sackler Career Development Chair for Young Faculty. Cofunded by the European Union (ERC *ReMembrane 101077502*). Views and opinions expressed are, however, those of the authors only and do not necessarily reflect those of the European Union or the European Research Council Executive Agency. Neither the European Union nor the granting authority can be held responsible for them. Li Yu was supported by the National Natural Science Foundation of China (grant nos. 32030023, 92354306, and 32330025), the Ministry of Science and Technology of the People's Republic of China (grant nos. 2024YFA1307301 and 2024YFF1502900), Tsinghua-Toyota Joint Research Fund (grant no. 20233930058), Scientific and Technological Innovation Project of China Academy of Chinese Medical Sciences (grant no. CI2023C024YL), and Tsinghua University Dushi Program (grant no. 20241080003).

AUTHOR CONTRIBUTIONS

D.W. and L.Y. conceived and planned the experimental studies of the hypotonically induced bulging in RFs of live cells. R.D. and R.S. conceived and planned the experimental studies of bulging in the tubular biomimetic system induced by membrane tension. B.Z. and M.M.K. conceived the theoretical studies. D.W. and R.D. performed the experiments. B.Z. performed the computations. R.S., L.Y., and M.M.K. supervised the work.

DECLARATION OF INTERESTS

L.Y. is the scientific founder of Migrasome Therapeutics Ltd. All other authors declare no competing interests related to this work.

SUPPORTING MATERIAL

Supporting material can be found online at https://doi.org/10.1016/j.bpj.2024.12.029.

REFERENCES

1. Ma, L., Y. Li, …, L. Yu. 2015. Discovery of the migrasome, an organelle mediating release of cytoplasmic contents during cell migration. *Cell Res.* 25:24–38.
2. Taylor, A. C., and E. Robbins. 1963. Observations on microextensions from the surface of isolated vertebrate cells. *Dev. Biol.* 6:660–673.
3. Bo, L., and R. E. Waugh. 1989. Determination of bilayer membrane bending stiffness by tether formation from giant, thin-walled vesicles. *Biophys. J.* 55:509–517.
4. Roux, A., G. Cappello, …, P. Bassereau. 2002. A minimal system allowing tubulation with molecular motors pulling on giant liposomes. *Proc. Natl. Acad. Sci. USA.* 99:5394–5399.
5. Derényi, I., F. Jülicher, and J. Prost. 2002. Formation and interaction of membrane tubes. *Phys. Rev. Lett.* 88:238101.
6. Jiang, D., Z. Jiang, …, L. Yu. 2019. Migrasomes provide regional cues for organ morphogenesis during zebrafish gastrulation. *Nat. Cell Biol.* 21:966–977.
7. Zhu, M., Q. Zou, …, L. Yu. 2021. Lateral transfer of mRNA and protein by migrasomes modifies the recipient cells. *Cell Res.* 31:237–240.
8. Jiao, H., D. Jiang, …, L. Yu. 2021. Mitocytosis, a migrasome-mediated mitochondrial quality-control process. *Cell.* 184:2896–2910.e13.
9. Qin, Y., L. Pan, …, W. Li. 2022. Pan-cancer analysis of AIM2 inflammasomes with potential implications for immunotherapy in human cancer: A bulk omics research and single cell sequencing validation. *Front. Immunol.* 13:998266.
10. Cheng, Y., J. Ren, …, Q. Zhang. 2022. Nanoparticulates reduce tumor cell migration through affinity interactions with extracellular migrasomes and retraction fibers. *Nanoscale Horiz.* 7:779–789.
11. Schmidt-Pogoda, A., J. K. Strecker, …, J. Minnerup. 2018. Dietary salt promotes ischemic brain injury and is associated with parenchymal migrasome formation. *PLoS One.* 13:e0209871.
12. Huang, Y., B. Zucker, …, L. Yu. 2019. Migrasome formation is mediated by assembly of micron-scale tetraspanin macrodomains. *Nat. Cell Biol.* 21:991–1002.
13. Rubinstein, E. 2011. The complexity of tetraspanins. *Biochem. Soc. Trans.* 39:501–505.
14. Dharan, R., Y. Huang, …, R. Sorkin. 2023. Tetraspanin 4 stabilizes membrane swellings and facilitates their maturation into migrasomes. *Nat. Commun.* 14:1037.
15. Sitarska, E., and A. Diz-Muñoz. 2020. Pay attention to membrane tension: Mechanobiology of the cell surface. *Curr. Opin. Cell Biol.* 66:11–18.
16. Wang Dongju, W. H., W. Wan, …, Z. Liu. 2024. Engineered Migrasomes: A Robust, Thermally Stable Vaccination Platform. *Elife.* 13:RP97621.
17. Gerstle, Z., R. Desai, and S. L. Veatch. 2018. Giant Plasma Membrane Vesicles: An Experimental Tool for Probing the Effects of Drugs and Other Conditions on Membrane Domain Stability. *Methods Enzymol.* 603:129–150.
18. Heinrich, V., and R. E. Waugh. 1996. A piconewton force transducer and its application to measurement of the bending stiffness of phospholipid membranes. *Ann. Biomed. Eng.* 24:595–605.
19. Cuvelier, D., I. Derényi, …, P. Nassoy. 2005. Coalescence of membrane tethers: experiments, theory, and applications. *Biophys. J.* 88:2714–2726.
20. Brakke, K. A. 1992. The Surface Evolver. *Exp. Math.* 1:141–165.
21. Helfrich, W. 1973. Elastic properties of lipid bilayers: theory and possible experiments. *Z. Naturforsch. C Biosci.* 28:693–703.
22. Oprea, J. 2007. Differential Geometry and its Applications. Mathematical Association of America, Washington, D.C.
23. Gibbs, J. W. 1961. The Scientific Papers. Dover, New York.
24. Zucker, B., G. Golani, and M. M. Kozlov. 2023. Model for ring closure in ER tubular network dynamics. *Biophys. J.* 122:1974–1984.
25. Kozlov, M. M. 2006. Some aspects of membrane elasticity. *In* Soft Condensed Matter Physics in Molecular and Cell Biology. W. C. K. Poon and D. Andelman, eds CRC press Taylor and Francis group, New York London, pp. 79–93.
26. Shi, Z., Z. T. Graber, …, A. E. Cohen. 2018. Cell Membranes Resist Flow. *Cell.* 175:1769–1779.e13.
27. Shi, Z., S. Innes-Gold, and A. E. Cohen. 2022. Membrane tension propagation couples axon growth and collateral branching. *Sci. Adv.* 8:eabo1297.
28. Mussel, M., and M. F. Schneider. 2021. Sound pulses in lipid membranes and their potential function in biology. *Prog. Biophys. Mol. Biol.* 162:101–110.
29. Bar-Ziv, R., T. Tlusty, …, A. Bershadsky. 1999. Pearling in cells: a clue to understanding cell shape. *Proc. Natl. Acad. Sci. USA.* 96:10140–10145.
30. Tsafrir, I., D. Sagi, …, J. Stavans. 2001. Pearling instabilities of membrane tubes with anchored polymers. *Phys. Rev. Lett.* 86:1138–1141.
31. Bar-Ziv, R., and E. Moses. 1994. Instability and "pearling" states produced in tubular membranes by competition of curvature and tension. *Phys. Rev. Lett.* 73:1392–1395.
32. Pullarkat, P. A., P. Dommersnes, …, A. Ott. 2006. Osmotically Driven Shape Transformations in Axons. *Phys. Rev. Lett.* 96:048104.
33. Zucker, B., and M. M. Kozlov. 2022. Mechanism of shaping membrane nanostructures of endoplasmic reticulum. *Proc. Natl. Acad. Sci. USA.* 119:e2116142119.
34. Heinrich, D., M. Ecke, …, G. Gerisch. 2014. Reversible membrane pearling in live cells upon destruction of the actin cortex. *Biophys. J.* 106:1079–1091.
35. Nelson, P., T. Powers, and U. Seifert. 1995. Dynamical Theory of the Pearling Instability in Cylindrical Vesicles. *Phys. Rev. Lett.* 74:3384–3387.
36. Ding, T., J. Ji, …, L. Yu. 2023. The phosphatidylinositol (4,5)-bisphosphate-Rab35 axis regulates migrasome formation. *Cell Res.* 33:617–627.
37. Haisha Liang, X. M., Y. Zhang, …, L. Yu. 2023. The formation of migrasomes is initiated by the assembly of sphingomyelin synthase 2 foci at the leading edge of migrating cells. *Nat. Cell Biol.* 25:1173–1184.

Biophysical Journal, Volume *124*

Supplemental information

Migrasome formation is initiated preferentially in tubular junctions by membrane tension

Ben Zucker, Raviv Dharan, Dongju Wang, Li Yu, Raya Sorkin, and Michael M. Kozlov

Supplementary Information

Supplementary Video legend

3-tube junction formation followed by swelling formation. A video of formation of 3-tube junction pulled from aspirated GPMV dyed with DiI-C12. Next the aspiration pressure was reduced to zero and then it was increased instantaneously to 0.39 mbar (corresponding to 0.074 mN/m). The video was composed of confocal fluorescence microscopy images of the experiment shown in Figure 1b. The fluorescence intensity is presented in logarithmic scale.

Supplementary Figure S1

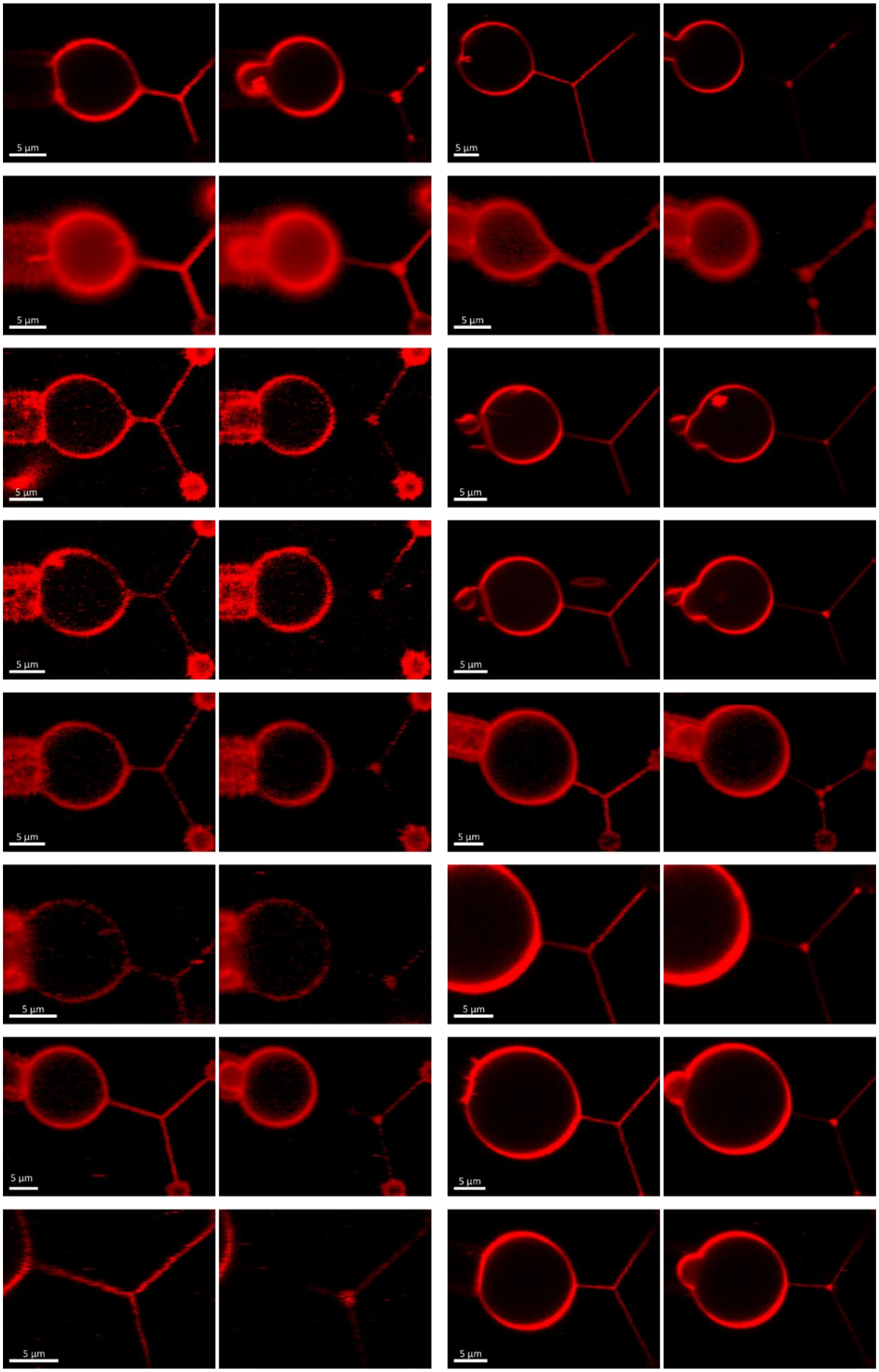

Figure S1. Confocal microscopy images of bulge formation induced by abrupt tension increase in 10 GPMVs dyed with DiI-C12. The images show 16 independent realizations of the system of three membrane tubules pulled out of an aspirated GPMV and connected by a three-way junction. In each panel the left and right images show, respectively, the system before and after the abrupt tension application. In all 16 experiments, the main bulging occurred in the junction.

Supplementary Information

Bulge coexistence in junction and tubular arms

The master curves obtained for the cylinder and the junction systems (Fig.7C of the main part) enable analysis of a mixed state of the junction system in which bulging occurs in parallel in the junction and in the cylindrical part of a tubular arm. Our goal here is to demonstrate that such coexistence of bulges represents a (quasi) equilibrium and to analyze the stability of this state with respect to bulging only in the junction or only in the tubular arm.

As above, in the initial state the junction is under a tension γ_0 and a vanishing pressure, $P_0 = 0$, the arm length, L_0, and the corresponding initial area, A_0, and volume, V_0. We assume L_0 to be so long that beginning from a distance L_j from the junction center the shape of each arm is unperturbed by the junction and, therefore, must behave as a one-cylindrical system. We consider the part of one of the arms spanned between L_j to L_0 as a one-cylindrical subsystem of the junction system.

We analyze a specific alteration of the whole system consisting in introduction of an extra volume, ΔV , and the following system evolution to the energetically preferable configuration by keeping constant the total area, A_0, the total volume, $V_0 + \Delta V$, and the total arm length, $3L_0$, The total volume addition, ΔV, is taken sufficiently large for driving the bulging transition.

We considered three possible scenarios: bulging only within the junction, bulging only within the one-cylindrical sub-system, parallel bulging in the junction and the one-cylinder sub-system. Our goal was to analyze the parameters and, specifically, the pressure, P, and the tension, γ of the energetically preferable configuration forming in each scenario as a function of the relative volume alteration, $\frac{\Delta V}{V_0}$. The scenario with the lowest pressure, P, for a given $\frac{\Delta V}{V_0}$ was considered as the optimal one.

For the scenario of bulging only in the junction the determination of the parameters of the energetically favorable configuration including the function $P\left(\frac{\Delta V}{V_0}\right)$ were determined according to the recipe described above (Eqs.21-25 of the main part) and based on the master curves for the junction system (Fig.7C red dots).

The procedure for the bulge coexistence scenario or only in the one-cylinder subsystem connected to a junction was a little more involved because of an apriori unknown distribution of the extra-volume ΔV and the related excess volume, V_{exc}, and area, A_{exc}, between the one-cylinder subsystem and the rest of the junction system. The solution in this case consisted in the following steps. We first assumed the excess values in the one-cylinder subsystem and the rest of the junction system to be $V_{exc}^{(cyl)}$, $A_{exc}^{(cyl)}$, and $V_{exc}^{(jun)}$, $A_{exc}^{(jun)}$, respectively. Using this assumption, we found the parameters of bulging, or the sub-bulging swelling, separately for the one-cylinder sub-system and in the rest of the junction system using the above protocols. Then we obtain all the four parameters, $V_{exc}^{(jun)}$, $A_{exc}^{(jun)}$, $V_{exc}^{(cyl)}$, $A_{exc}^{(cyl)}$ as functions of $\frac{\Delta V}{V_0}$ by using the conditions of conservation of the total volume and area of the whole system and the conditions of equal pressure, P, and tension, γ, in the one-cylinder sub-system and the rest of the junction system. The essence of the condition of the pressure and tension equality is the thermodynamic equilibrium between the one-cylider sub-system and the rest of the junction system and, hence, the (quasi) equilibrium of the whole junction system.

It was possible to obtain the numerical solution for the parameters of the coexistence scenarios meaning that the coexistence of bulges in the junction and tubular arm can indeed be a (quasi)equilibrium state of the system.

The computed dependences of pressure on the relative added volume, $P\left(\frac{\Delta V}{V_0}\right)$, are presented for all the scenarios in (Fig.S2A), which shows that bulging in junction only is characterized by the lowest pressure and, hence, must be the most energetically favorable.

In addition, our computations enabled determination of the partitioning of the system's volume between the bulges and the tubular regions, whose results are presented in (Fig.S2 B-D). Finally, the computed conformation of the quasi-equilibrium configuration of coexisting bulges is presented in (Fig.S2 E).

Figure S2

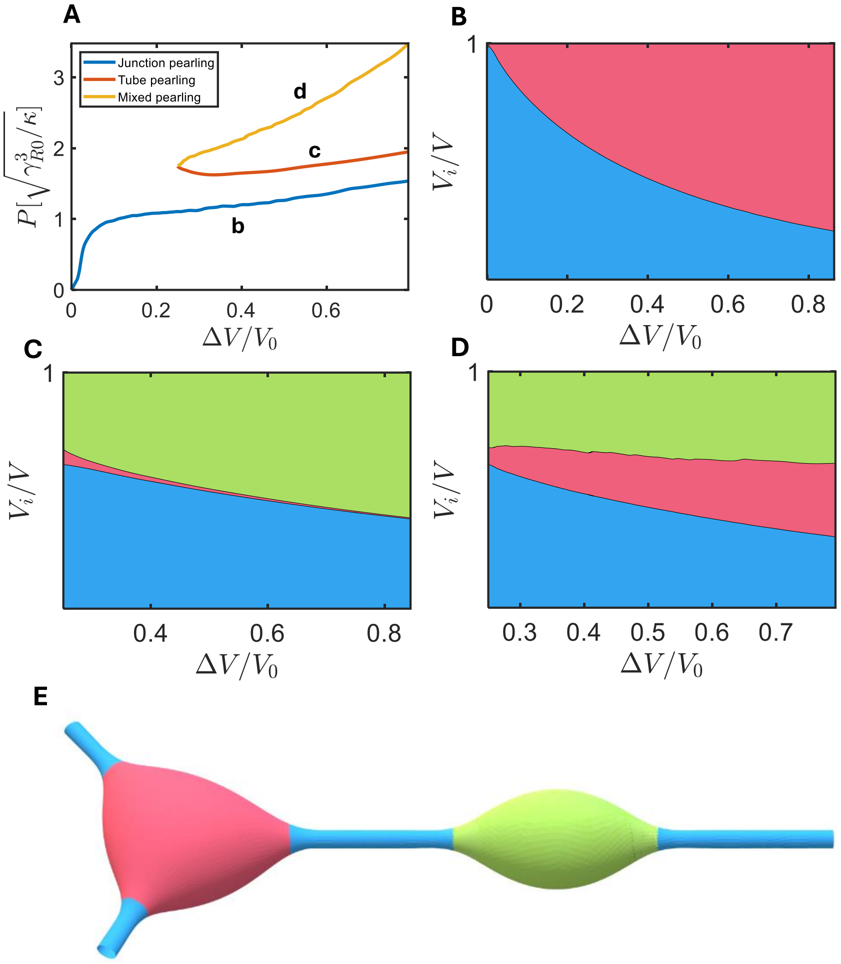

Figure S2. Comparison of bulging scenarios. Computational examination of three scenarios of bulging. (A) The dimensionless pressure, P, of the energetically favorable configurations computed for bulging of the junction (blue curve), bulging of the tubular arm (orange curve), bulging of both the junction and the tubular arm (yellow curve) as functions of the normalized extra volume, $\frac{\Delta V}{V_0}$, where V_0 is the initial volume

of the system. (B-D) Partitioning of volume between different parts of the system in dependence on $\frac{\Delta V}{V_0}$. The volume fraction is denoted by $\frac{V_i}{V}$. The blue, red and green colors correspond respectively to the tubular arms, junctional bulge, and tubular bulge. (B) Bulge in junction. (C) Bulge in tubular arm. (D) Coexistence of bulges in the junction and tubular arm. (E) Example of a computed conformation of the state of coexisting bulges. The length of the tubular network for (A-D) is 16 λ_0.

References

(1) Zucker, B.; Golani, G.; Kozlov, M. M. Model for ring closure in ER tubular network dynamics. *Biophys J* **2023**, *122* (11), 1974-1984. DOI: 10.1016/j.bpj.2022.10.005 From NLM Medline.

(2) Kozlov, M. M. Some aspects of membrane elasticity. In *Soft condensed matter physics in molecular and cell biology*, Poon, W. C. K., Andelman, D. Eds.; Scottish Graduate Series, CRC press, Taylor and Francis group, 2006; pp 79-93.

(3) Gibbs, J. W. *The scientific papers*; Dover, 1961.

(4) Helfrich, W. Elastic properties of lipid bilayers: theory and possible experiments. *Z Naturforsch C* **1973**, *28* (11), 693-703. DOI: 10.1515/znc-1973-11-1209 From NLM Medline.

(5) Brakke, K. A. The Surface Evolver. *Experimental Mathematics* **1992**, *1* (2), 141-165. DOI: 10.1080/10586458.1992.10504253.
